# Comparative transcriptomics of two *Salvia* subg. *Perovskia* species contribute towards molecular background of abietane-type diterpenoid biosynthesis

**DOI:** 10.1038/s41598-024-53510-5

**Published:** 2024-02-06

**Authors:** Monika Bielecka, Marta Stafiniak, Bartosz Pencakowski, Sylwester Ślusarczyk, Jan Paweł Jastrzębski, Łukasz Paukszto, Łukasz Łaczmański, Shima Gharibi, Adam Matkowski

**Affiliations:** 1 Department of Pharmaceutical Biology and Biotechnology, Wroclaw Medical University, Borowska 211A, 50-556 Wrocław, Poland; 2 Department of Plant Physiology, Genetics and Biotechnology, Faculty of Biology and Biotechnology, University of Warmia and Mazury in Olsztyn, Oczapowskiego 1A/113, 10-719 Olsztyn, Poland; 3 Department of Botany and Nature Protection, Faculty of Biology and Biotechnology, University of Warmia and Mazury in Olsztyn, Prawocheńskiego 17, 10-720 Olsztyn, Poland; 4 Laboratory of Genomics & Bioinformatics, Hirszfeld Institute of Immunology and Experimental Therapy PAS, Rudolfa Weigla 12, Wrocław, Poland; 5 Core Research Facilities (CRF), Isfahan University of Medical Sciences, Isfahan, 81746-73461 Iran; 6 Botanical Garden of Medicinal Plants, Wroclaw Medical University, Jana Kochanowskiego 14, Wrocław, Poland

**Keywords:** Natural variation in plants, Plant molecular biology, Plant physiology, Secondary metabolism

## Abstract

Tanshinones, are a group of diterpenoid red pigments present in Danshen – an important herbal drug of Traditional Chinese Medicine which is a dried root of *Salvia miltiorrhiza* Bunge. Some of the tanshinones are sought after as pharmacologically active natural products. To date, the biosynthetic pathway of tanshinones has been only partially elucidated. These compounds are also present in some of the other *Salvia* species, i.a. from subgenus *Perovskia*, such as *S. abrotanoides* (Kar.) Sytsma and *S. yangii* B.T. Drew. Despite of the close genetic relationship between these species, significant qualitative differences in their diterpenoid profile have been discovered. In this work, we have used the Liquid Chromatography–Mass Spectrometry analysis to follow the content of diterpenoids during the vegetation season, which confirmed our previous observations of a diverse diterpenoid profile. As metabolic differences are reflected in different transcript profile of a species or tissues, we used metabolomics-guided transcriptomic approach to select candidate genes, which expression possibly led to observed chemical differences. Using an RNA-sequencing technology we have sequenced and de novo assembled transcriptomes of leaves and roots of *S. abrotanoides* and *S. yangii*. As a result, 134,443 transcripts were annotated by UniProt and 56,693 of them were assigned as Viridiplantae. In order to seek for differences, the differential expression analysis was performed, which revealed that 463, 362, 922 and 835 genes indicated changes in expression in four comparisons. GO enrichment analysis and KEGG functional analysis of selected DEGs were performed. The homology and expression of two gene families, associated with downstream steps of tanshinone and carnosic acid biosynthesis were studied, namely: cytochromes P-450 and 2-oxoglutarate-dependend dioxygenases. Additionally, BLAST analysis revealed existence of 39 different transcripts related to abietane diterpenoid biosynthesis in transcriptomes of *S. abrotanoides* and *S. yangii*. We have used quantitative real-time RT-PCR analysis of selected candidate genes, to follow their expression levels over the vegetative season. A hypothesis of an existence of a multifunctional CYP76AH89 in transcriptomes of *S. abrotanoides* and *S. yangii* is discussed and potential roles of other CYP450 homologs are speculated. By using the comparative transcriptomic approach, we have generated a dataset of candidate genes which provides a valuable resource for further elucidation of tanshinone biosynthesis. In a long run, our investigation may lead to optimization of diterpenoid profile in *S. abrotanoides* and *S. yangii*, which may become an alternative source of tanshinones for further research on their bioactivity and pharmacological therapy.

## Introduction

Subgenus *Perovskia* of the extended genus of *Salvia* comprises several Central Asian medicinal and aromatic species, of which *Salvia abrotanoides* (Kar.) Sytsma and *S. yangii* B.T. Drew are the most widespread. Commonly known as Caspian Russian sage, *S. abrotanoides* is native for Central and Northeastern Iran, Northern Pakistan and Northwestern India^1^. S*. yangii*, with a common name Russian sage, grows in Afghanistan, Eastern Iran, Pakistan, Tibet and Xinjiang in China^2–4^.

Both species are used as folk medicinal herbs, the application history differs, however, due to their distribution area^5^. *S. abrotanoides*, locally known as brazambol, is used for the treatment of typhoid, fever, headache, toothache, gonorrhoea, vomiting, cardiovascular diseases, liver fibrosis, painful urination and cough and as a sedative, analgesic and antiseptic drug^6–10^. *S. abrotanoides* was also proved to have cytotoxic, antiplasmodial and antiinflammatory activities that support its use to treat leishmaniasis in Iranian traditional medicine^11,12^. *S. yangii* is used as a cooling medicine in the treatment of fever, dysentery, scabies, diabetes, and as an antibacterial remedy to heal wounds^5,6,13–15^. In traditional Tibetan and Chinese medicine, it has been claimed that *S. yangii* is a powerful analgesic and parasiticide agent^16,17^.

*S. abrotanoides* and *S. yangii* display a wide array of bioactive natural compounds belonging to different chemical classes^5^. Apart from essential oil constituents^18–21^ and phenolic compounds^1,22–25^, they produce also a wide range of abietane-type diterpenoids. Aerial parts of *S. abrotanoides* and *S. yangii* contain carnosic acid, rosmanol and their derivatives^26–28^. Roots of *S. abrotanoides* and *S. yangii* are abundant in red-colored quinoid nor-abietanoids collectively called tanshinones. Tanshinones are the main constituents of the important Traditional Chinese Medicine herbal medicine – ‘Danshen’ (*Salviae miltiorrhizae rhizoma et radix* – a pharmacopoeial drug of China and Europe)^29–32^. Hence, roots of *S. abrotanoides* and *S. yangii* can be considered an alternative source of these pharmacologically active compounds.

To date, a total of 81 different tanshinones were identified^33^. Multiple pharmacological studies have proven that tanshinones possess various biological activities including antibacterial, antioxidant, anti-inflammatory and anti-tumor effects^34–36^. Due to the structural uniqueness of tanshinones, their biosynthesis has been studied intensively in the last two decades, in several species of *Lamiaceae*, incuding: *S.miltiorrhiza*^30,33,37–41^, *Salvia rosmarinus*^26,42^, *Salvia pomifera*^43^, *Salvia fruticosa*^26^ or *Salvia apiana*^44^. Recently, a comprehensive, in-depth review of the tanshinone biosynthesis has been published^45^. However, the biosynthetic pathway of tanshinones is only partially elucidated. It is known that tanshinones originate from the universal isoprenoid precursors isopentenyl diphosphate (IPP) and dimethylallyl diphosphate (DMAPP) produced via cytoplasmic mevalonate (MVA) pathway and (to a larger extend) in the plastidic 2-C-methyl-D-erythritol 4-phosphate (MEP) pathway^46^. DMAPP and three units of IPP are then being condensed to form the general diterpenoid precursor (E,E,E)-geranylgeranyl diphosphate (GGPP) by a GGPP synthase (GGPPS). GGPP undergoes cyclization catalyzed by a class II diterpene cyclase, represented in *S. miltiorrhiza* by SmCPS1, to form copalyl diphosphate (CPP). In a further cyclization and/or rearrangement catalyzed by a class I diterpene cyclase, encoded in *S. miltiorrhiza* by SmKSL1, CPP is converted to a tricyclic abietane-type backbone, called miltiradiene^47,48^. In the presence of oxygen, miltiradiene undergoes a spontaneous aromatization of its C-ring into an abietatriene, which is then hydroxylated at C12 by a CYP76AH subfamily, represented in *S. miltiorrhiza* by CYP76AH1, to form ferruginol^49–51^. Subsequential oxygenation of ferruginol is catalysed in *S. miltiorrhiza* by CYP76AH3 and CYP76AK1. Since SmCYP76AH3 and SmCYP76AK1 were shown to present substrate promiscuity by accepting a range of different substrates or oxidizing their substrates in various positions, including multiple oxidation steps and thus yielding an array of oxidation products, a bifurcating pathway of tanshinone biosynthesis was speculated. However, it is most likely that *in planta* further oxygenation of ferruginol in *S. miltiorrhiza* is catalysed sequentially by CYP76AH3, acting as 11-hydroxylase, and later by CYP76AK1 acting as a 20-hydroxylase^52^. The next step, which requires demethylation (loss of C20) and aromatization of ring B to form miltirone, remains unresolved. Next, two CYP71D enzymes (CYP71D375 and CYP71D373) were characterized in *S. miltiorrhiza* to catalyze the D-ring formation through C16 hydroxylation and 14,16-ether (hetero) cyclization, leading to the formation of dihydrofuran-tanshinones from their respective precursors, including cryptotanshinone^53^. Recently, a 2-oxoglutarate-dependent dioxygenase from *S. miltiorrhiza* (Sm2-ODD14) has been shown to act as a dehydrogenase catalyzing the furan ring formation for the tetracyclic tanshinone backbone. In vitro Sm2-ODD14 converted cryptotanshinone to tanshinone IIA (TIIA) and thus was designated TIIA synthase (SmTIIAS)^33^. Although numerous genome sequencing and comparative omics experiments has been performed to yield several promising cytochromes P450s, 2-ODD and short chain alcohol dehydrogenase (SDR) candidates that may possibly complete the final steps in the tanshinone biosynthetic pathway, it is still unclear which transformations lead to the biosynthesis of the wide array of tanshinones^40,45,54^.

*S. rosmarinus* and few other sage species also produce another important abietane diterpenoid – carnosic acid^55,56^. In these plants, enzymes orthologous to CYP76AH3 such as CYP76AH4 and CYP76AH22‐24 are also able to produce 11‐hydroxy ferruginol. Next, three sequential C20 oxidations catalyzed by CYP76AK6‐8 convert 11‐hydroxy ferruginol to carnosic acid, which spontaneously oxidizes to carnosol^26,57,58^.

Recently, we have performed a targeted comparison of the metabolic profiles of *S. abrotanoides* and *S. yangii*, which revealed differences between these two species, despite their close taxonomic relationship^28^. The analysis of leaves showed the presence of 13 different diterpenoids in *S. yangii*, while *S. abrotanoides* contained 15 diterpenoids, out of which carnosic acid quinone, isorosmanol, and trilobinol were exclusively in *S. abrotanoides*. On the other hand, rosmaridiphenol and sugiol were detected only in *S. yangii* leaves. In the roots, diterpenoids were represented by 18 compounds in *S. yangii* and 13 in *S. abrotanoides* of which twelve were common, including the most abundant cryptotanshinone. Six diterpenoids were unique for *S. yangii*: didehydrotanshinone IIa, acetyloxycryptotanshinone, isograndifoliol, grandifoliol, didehydroacetyloxycryptotanshinone, and ketoisograndifoliol. An unidentified cryptotanshinone derivative was found exclusively in *S. abrotanoides* roots. Out of nine compounds which were not determined in methanolic extracts of roots, three were detected only in *S. yangii*.

Following our previous investigations, we have performed a quantitative analysis of abietane diterpenoids in leaves and roots of *S. abrotanoides* and *S. yangii* during the vegetative season. We also used Illumina next generation sequencing (NGS) with de novo transcript assembly to obtain the transcriptomes of leaves and roots of *S. abrotanoides* and *S. yangii* and identified genes encoding for orthologous abietane diterpenoid-related enzymes in these species together with their transcriptional pattern. Having access to two closely related, yet separate, species of different abietane diterpenoid profiles in leaves and roots, we used comparative analysis of their transcriptomes to select candidate genes, which expression possibly led to observed metabolic differences. By using the metabolomics-guided transcriptomic approach we aimed at discovering candidate genes potentially involved in the biosynthesis of abietane diterpenoids, including tanshinones.

## Results

### Analysis of diterpenoids during the vegetation season

Out of 15 different abietane diterpenoids detected earlier in leaves of *S. abrotanoides* and 13 in leaves of *S. yangii*, ten were estimated quantitatively, including: carnosic acid (**1**), carnosol (**2**), rosmanol (**3**) and their isoforms and derivatives as well as sugiol (**9**) and trilobinol (**10**). Using UHPLC-qTOF-MS, 13 and 18 different diterpenoids were found in the roots of *S. abrotanoides* and *S. yangii*, respectively^28^. Out of these, eight compounds were estimated quantitatively: cryptotanshinone (**11**) with its three isoforms and derivatives, didehydrotanshinone IIA (**18**), OH-tanshindiol A (**17**), isograndifoliol (**15**) and ketoisograndifoliol (**16**).

The number of abietane diterpenoid compounds differed between organs and species (Table 1). In leaves, carnosol (**2**) was the most abundant compound in both species, yielding 2.32 mg/g d.w. and 2.17 mg/g d.w. at the start of the season (SOS) in *S. abrotanoides* and *S. yangii*, repectively. The amount of carnosol was peaking at the end of season (EOS) in *S. abrotanoides* (3.11 mg/g d.w.), while in *S. yangii* it was clearly decreasing towards the end of the season (0.95 mg/g d.w.). In the contrary, the carnosol precursor – carnosic acid (**1**) was undetectable in the leaves of *S. abrotanoides* or at the limit of detection in the leaves of *S. yangii*. Relatively low amounts of rosmanol (**3**) were present in the middle of the season (MOS) in the leaves of both species, equal to 0.68 and 0.15 mg/g d.w. of *S. abrotanoides* and *S. yangii*, repectively. At the start and the end of the season, rosmanol was not or hardly detectable. Interestingly, its derivative, the 11,12-dimethylrosmanol (**6**) was present at 3.17 mg/g d.w. in the leaves of *S. abrotanoides* at the start of the season, which was significantly higher than in the middle and at the end of the season as well as in the leaves of *S. yangii*. Other two rosmanol derivatives: 7-methylrosmanol (**8**) and 11-methylrosmanol (**7**) as well as epi-rosmanol (**4**) were accumulating in the middle of the season (MOS), and their amounts in the leaves of *S. abrotanoides* were significantly higher than in *S. yangii*. The highest amounts of isorosmanol (**5**) were detected at the start of the season (SOS) in the leaves of both species. Isorosmanol level was relatively stable throughout the vegetative season in *S. abrotanoides* while in *S. yangii* it decreased. Sugiol (**9**) was detected exclusively in the leaves of *S. yangii*, whereas trilobinol (**10**) was unique for *S. abrotanoides*, both peaking in spring.

**Table 1:** The content of abietane diterpenoids [mg/g d.w. ± SD, n = 3] in leaves and roots of *S. abrotanoides* and *S. yangii*, at the start of the vegetation season (SOS), middle of the season (MOS) and end of the season (EOS).

No	Compound	*Salvia abrotanoides*			*Salvia yangii*		
		SOS	MOS	EOS	SOS	MOS	EOS
		Leaves					
**1**	Carnosic acid	0.00	0.00	0.00	0.09 ± 0.01^c^	0.05 ± 0.01^a^^,^^c^	0.01 ± 0.00^a^^,^^b^
**2**	Carnosol	2.32 ± 0.79	1.48 ± 0.38	3.11 ± 1.41	2.17 ± 0.19	1.19 ± 0.19	0.95 ± 0.08^c^
**3**	Rosmanol	0.01 ± 0.00	0.68 ± 0.27^a^	0.05 ± 0.01^*b*^	0.00	0.15 ± 0.03^*c*^	0.00
**4**	Epi-rosmanol	0.02 ± 0.01	1.42 ± 0.08^a^	0.07 ± 0.05^b^	0.08 ± 0.03	0.35 ± 0.07^*a*^^,^^c^	0.03 ± 0.00^b^
**5**	Isorosmanol	0.49 ± 0.19	0.33 ± 0.08	0.41 ± 0.30	0.58 ± 0.07	0.02 ± 0.00^a^	0.13 ± 0.01^a^
**6**	11,12-dimethylrosmanol	3.17 ± 0.17	0.05 ± 0.01^a^	0.76 ± 0.36^a^	0.11 ± 0.03^c^	0.00	0.11 ± 0.02^b,c^
**7**	11-methylrosmanol	0.04 ± 0.02	0.97 ± 0.42^*a*^	0.08 ± 0.05^*b*^	0.16 ± 0.02	0.25 ± 0.05^c^	0.08 ± 0.01
**8**	7-methylrosmanol	0.15 ± 0.11	2.44 ± 0.74^a^	0.15 ± 0.09^b^	0.57 ± 0.12	0.88 ± 0.27^*c*^	0.07 ± 0.01
**9**	Sugiol	0.00	0.00	0.00	1.67 ± 0.11^c^	0.59 ± 0.02^a^^,^^c^	0.96 ± 0.10^a^^,^^b^^,^^c^
**10**	Trilobinol	0.80 ± 0.14	0.42 ± 0.05^a^	0.66 ± 0.25	0.00^c^	0.00^c^	0.00^*c*^
		Roots					
**11**	Cryptotanshinone	5.06 ± 0.47	7.51 ± 0.92	9.89 ± 0.53^*a*^	7.17 ± 0.97	9.12 ± 1.35	7.91 ± 1.11
**12**	OH-cryptotanshinone	0.39 ± 0.06	1.25 ± 0.19^a^	1.61 ± 0.05^a^	2.92 ± 0.25^c^	3.94 ± 0.36^a,^^c^	3.57 ± 0.48^c^
**13**	Oxocryptotanshinone	0.26 ± 0.02	0.31 ± 0.09	0.54 ± 0.12	1.01 ± 0.28^*c*^	0.49 ± 0.04^a^	1.00 ± 0.06^b,c^
**14**	Acetyloxycryptotanshinone	0.00	0.00	0.00	0.52 ± 0.03^c^	1.41 ± 0.18^a^^,^^c^	1.33 ± 0.10^a^^,^^c^
**15**	Isograndifoliol	0.00	0.00	0.84 ± 0.10	0.75 ± 0.45	4.22 ± 0.59^a^^,^^c^	4.73 ± 0.50^a^^,^^c^
**16**	Ketoisograndifoliol	0.00	0.00	0.00	0.00	0.55 ± 0.14^a^^,^^c^	0.68 ± 0.08^a^^,^^c^
**17**	OH-tanshindiol A	0.02 ± 0.00	0.09 ± 0.05	0.05 ± 0.00	0.29 ± 0.02^c^	0.42 ± 0.07^a,^^c^	0.31 ± 0.01^b,c^
**18**	Didehydrotanshinone IIA	0.00	0.00	0.00	2.33 ± 0.41^c^	2.92 ± 0.48^c^	2.40 ± 0.32^c^

Statistical significance of differences in chemical parameters between samples was evaluated with a one-way ANOVA with post hoc Tukey’s multiple comparison tests.

^a^Significant differences in comparison to SOS; ^b^Significant differences in comparison to MOS; ^c^Significant differences in comparison to *S. abrotanoides*; The level of significance is indicated by the used fonts: p < 0.01 lower case font, p < 0.001 italic font, p < 0.0001 underlined font; *SOS* the start of the season, *MOS* the middle of the season, *EOS* the end of the season.

**Figure 1:**
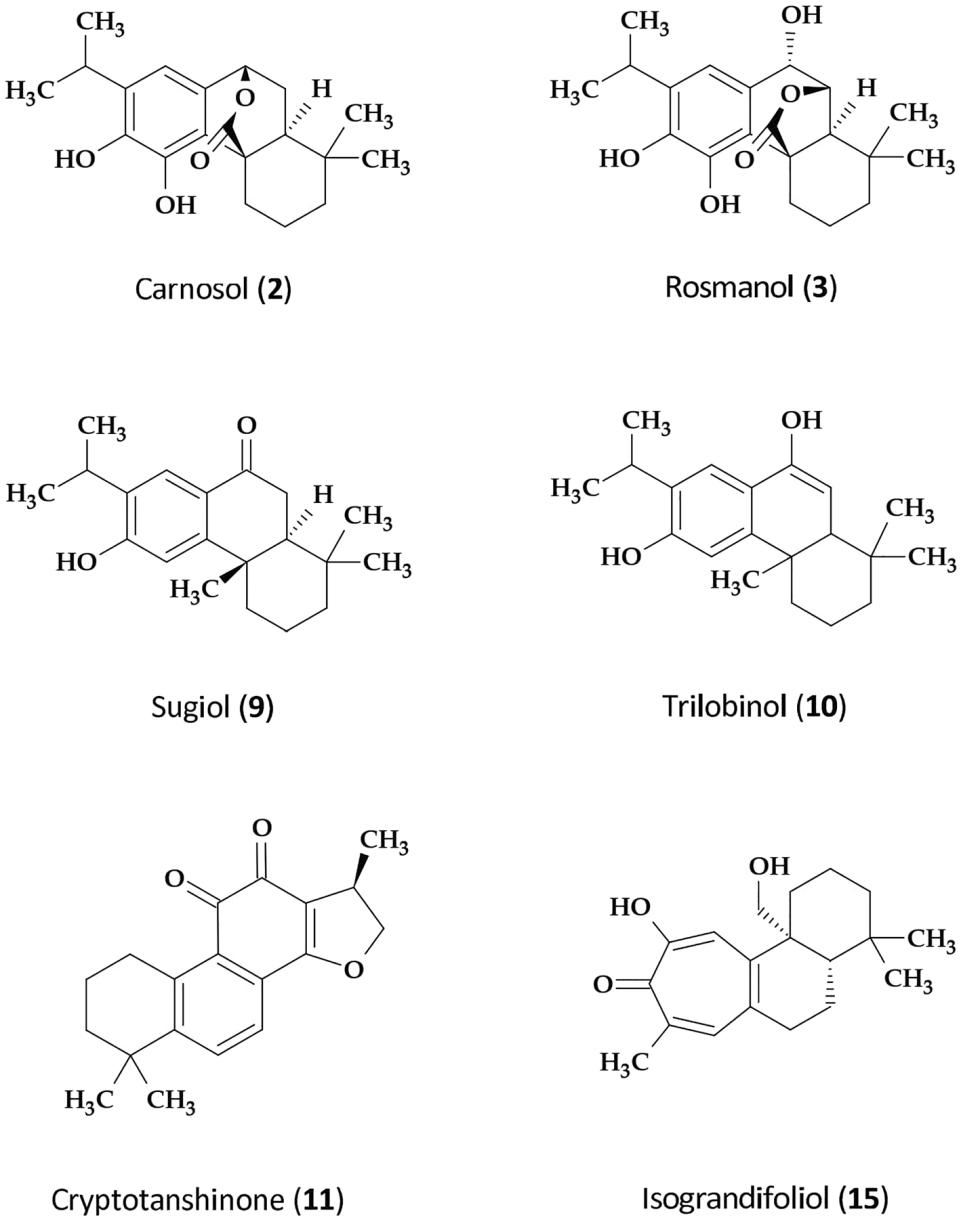
Chemical structures of the most abundant or unique abietane diterpenoids detected in *S. abrotanoides* and *S. yangii*.

Quantitative analysis showed that the diterpenoid profile of in roots was clearly dominated by cryptotanshinone (**11**) reaching 9.89 mg/g d.w. at the EOS in *S. abrotanoides* and 9.12 mg/g d.w. in the MOS in *S. yangii* (Table 1). A similar pattern of accumulation was observed for its derivatives: OH-cryptotanshinone (**12**) and oxocryptotanshinone (**13**), although their amounts were lower than cryptotanshinone. Second in terms of abundance, was isograndifoliol (**15**), which was initially thought to be exclusive for the roots of *S. yangii*^28^. Although the level of isograndifoliol remained undetectable in the roots of *S. abrotanoides* at the start and in the middle of the season, it occurred in the autumn at 0.84 mg/g d.w.. Conversely, higher levels of isograndifoliol were detected in *S. yangii*, in the middle and at the end of the season, reaching 4.22 and 4.73 mg/g d.w., respectively. The content of OH-tanshindiol A (**17**) was significantly higher in the roots of *S. yangii* than in *S. abrotanoides*, throughout the whole season, peaking during flowering (MOS) with 0.42 mg/g d.w. Didehydrotanshinone IIA (**18**), acetyloxycryptotanshinone (**14**) and ketoisograndifoliol (**16**) were unique for *S. yangii*. Relatively stable and high amounts of the didehydrotanshinone IIA were noted throughout the season, reaching 2.92 mg/g d.w. in the roots of *S. yangii* in the MOS. Also, the acetyloxycryptotanshinone accumulated in the MOS (1.41 mg/g d.w.), while the highest level of ketoisograndifoliol was at the EOS (0.68 mg/g d.w.) in *S. yangii* roots. Although the presence of tanshinone IIA, the main constituent of Danshen, was detected in roots of both *S. abrotanoides* and *S. yangii*^28^, its low amounts did not allow to analyse its content quantitatively. The chemical structures of the most abundant or unique abietane diterpenoids detected in *S. abrotanoides* and *S. yangii* are presented in Fig. 1.

### RNA-sequencing, de novo transcriptome assembly and transcript functional annotation

**Figure 2:**
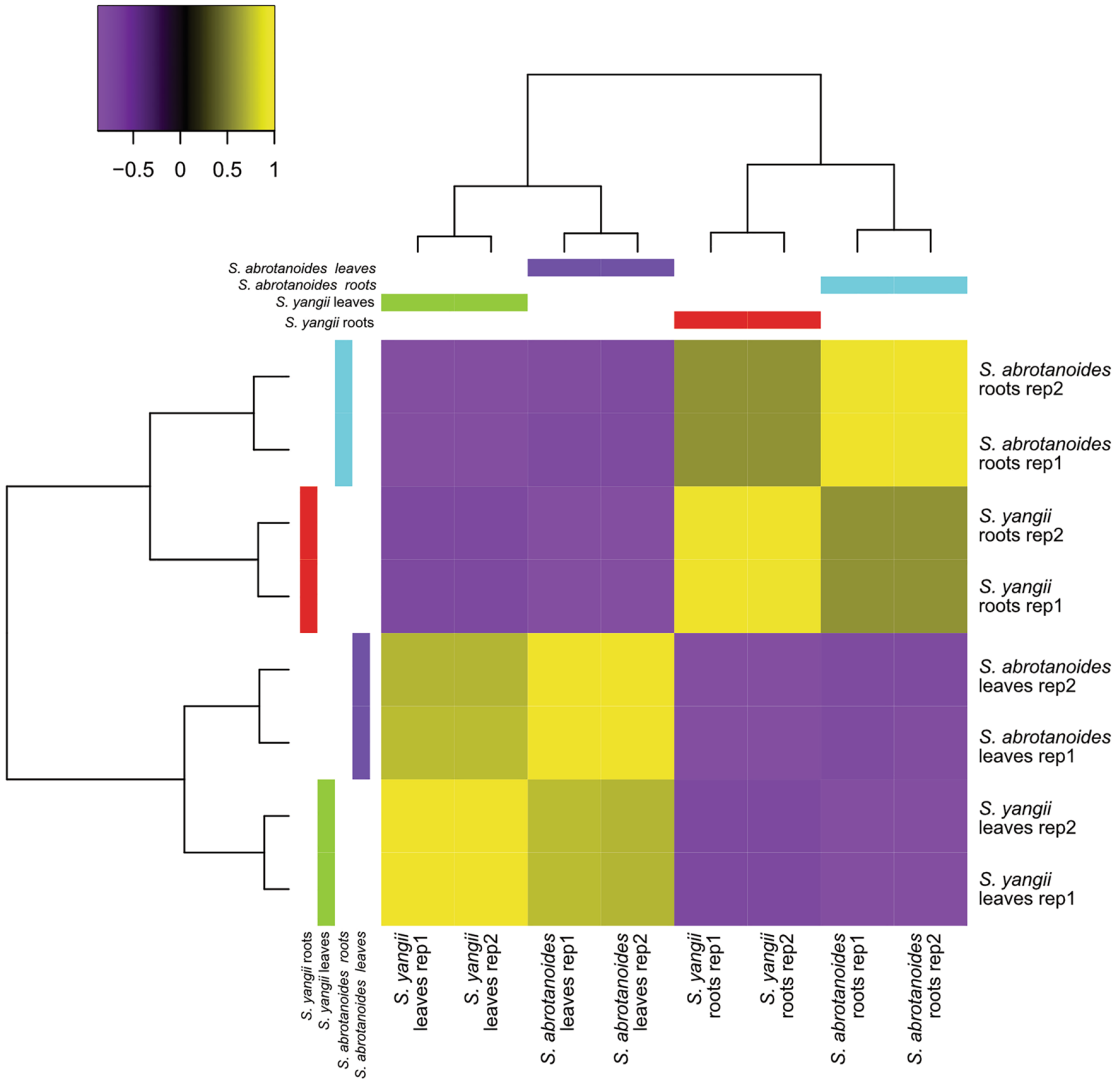
Overall expression profiles of biological replicates extracted from roots and leaves of *S. abrotanoides* and *S. yangii*. The phenomap clusters created according correlation of global samples expression.

High-throughput sequencing generated over 60 million reads for each cDNA library. After quality control process, the range of 52.90—59.34 million of trimmed reads passed to filtration procedure (Supplementary Tables S1 and S2). Next, to avoid creation of chimeric transcripts, reads were divided according to species affiliation and two de novo processes were performed. De novo assembly has generated 372,862 potential transcripts for *S. yangii* and 258,647 for *S. abrotanoides*. Gene identification revealed presence of 93,610 potential protein encoding transcripts in *S. yangii* and 155,739 in *S. abrotanoides*. More than 99% of the aforementioned core eukaryota genes predicted by BUSCO (complete and fragmented) confirmed the completeness of the assembly of both transcriptomes. Both transcriptomes were compared, and the identity of 1000 random homological sequences was tested, in the result 831 of them showed more than 97% of pairwise identity. Due to the high similarity of both transcriptomes, *S. abrotanoides* transcriptome was selected as a reference for further expression profiling. The 134,443 transcripts had UniProt annotation and 56,693 of them were assigned as *Viridiplantae*. The trimmed reads were remapped to the reference transcriptome (*S. abrotanoides*) and the overall library expression profiles were clustered according to plant organs, not by the species (Fig. 2), proving them suitable for subsequent comparative analysis between species.

### Comparative transcriptomic analysis to identify differentially expressed genes

**Figure 3:**
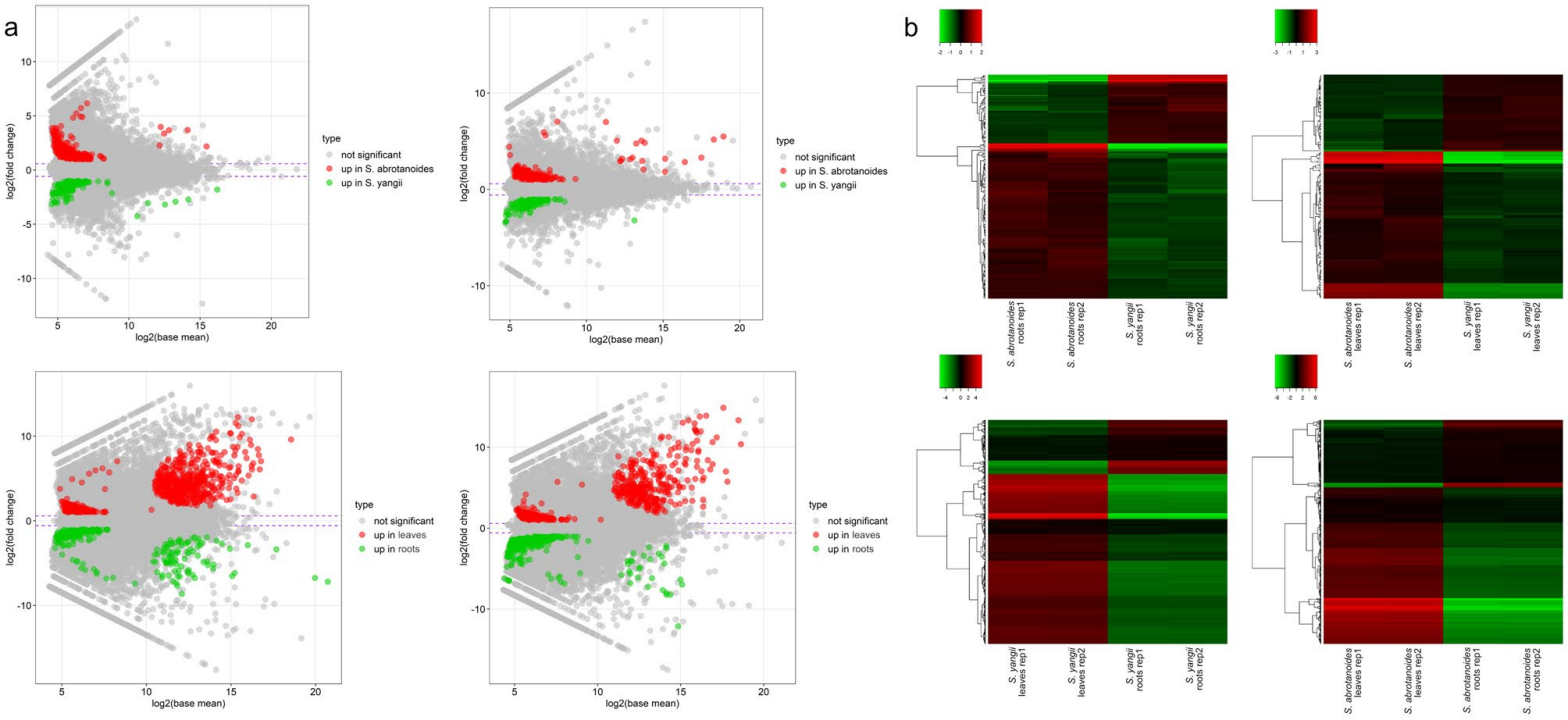
Selection of differentially expressed genes involved in four comparisons: *S. abrotanoides* roots versus *S. yangii* roots (upper left)*, S. abrotanoides* leaves versus *S. yangii* leaves (upper right), *S. yangii* roots versus leaves (lower left); and *S. abrotanoides* roots versus leaves (lower right). (**a**) MA plots of DEGs. Red and green dots depict DEGs significantly changing expression between plant organs and both species. (**b**) Heatmaps of significant DEGs with fluctuated expression. Expression values are Z-score scaled.

**Figure 4:**
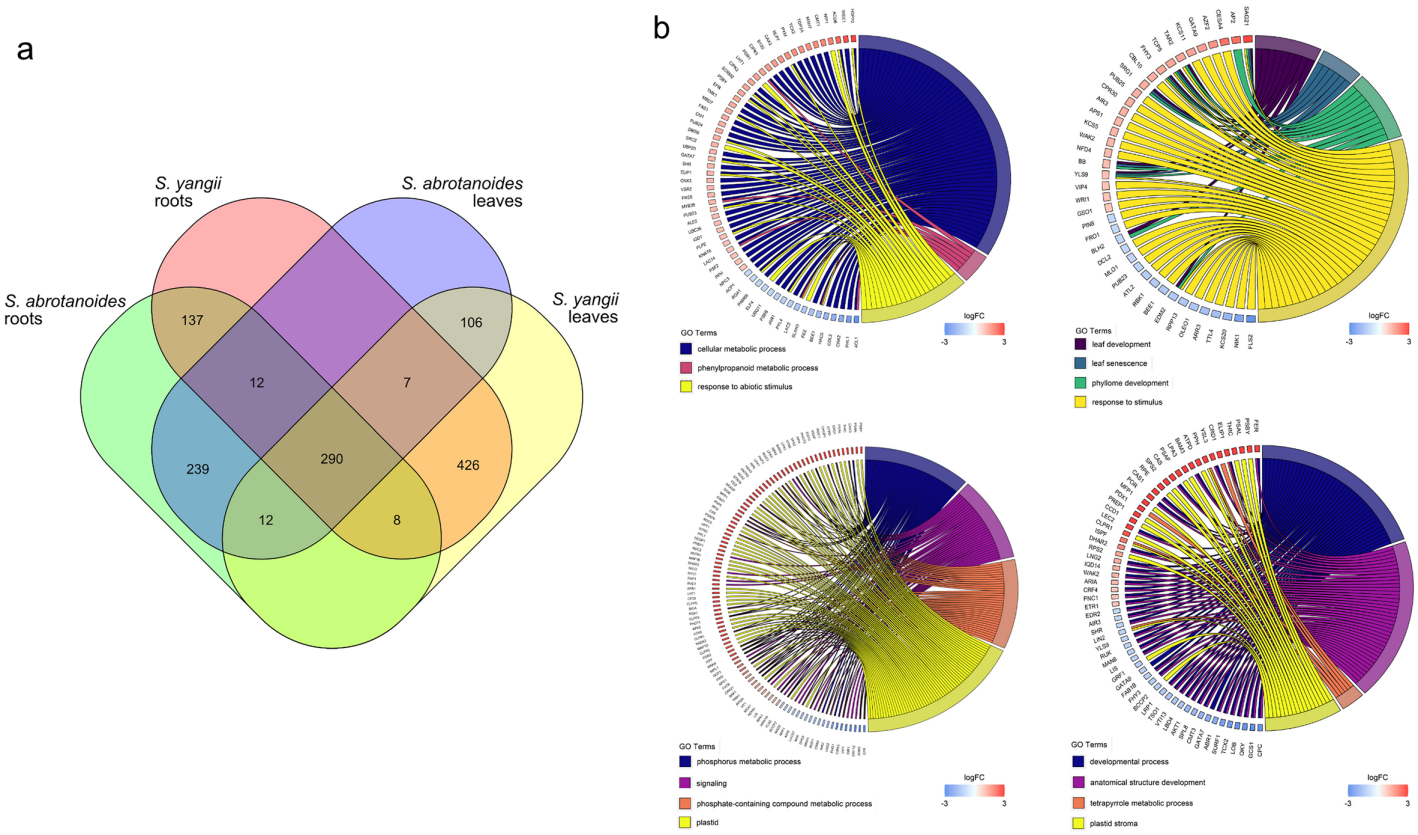
Visualization of differentially expressed genes. (**a**) Venn diagram of DEGs involved in four experimental comparisons. Numbers inside the blocks indicate DEGs assigned to particular plant organs in *Salvia* species. (**b**) Circular visualization of selected Gene Ontology terms and related DEGs in four experimental comparisons: *S. abrotanoides* roots versus *S. yangii* roots (upper left)*, S. abrotanoides* leaves versus *S. yangii* leaves (upper right), *S. yangii* roots versus leaves (lower left); and *S. abrotanoides* roots versus leaves (lower right).

The differential expression analysis consisted of four comparisons. 463, 362, 922 and 835 genes indicated changes in expression in following comparisons: *S. abrotanoides* roots versus* S. yangii* roots*, S. abrotanoides* leaves versus* S. yangii* leaves, *Salvia yangii* roots versus leaves; and *Salvia abrotanoides* roots versus leaves, respectively (Fig. 3a). In details, the 309 genes were expressed at significantly higher level in *S. abrotanoides* roots, and 154 protein coding transcripts had significantly higher expression in *S. yangii* roots. Likewise, the expression profiling of leaves showed 231 and 131 transcripts expressed at significantly higher level in *S. abrotanoides* and in *S. yangii*, respectively*.* Comparison between leaves and roots revealed 630 and 292 highly expressed DEGs, respectively in *S. yangii*, whereas in *S. abrotanoides,* the number of DEGs with higher expression was 479 in leaves and 356 in the roots. All selected DEGs are listed in Supplementary Table S3. When presented as heatmaps, significant DEGs showed fluctuated expression in all four comparisons (Fig. 3b). Additional comparative analysis showed that 290 DEGs were assigned to all four comparisons. Conversely, 106 DEGs were unique for leaves and 137 DEGs were significant only in root RNA of both species (Fig. 4a).

To figure out the most significant functional difference between analysed organs and species, the Gene Ontology (GO) enrichment of DEGs was further performed. Enrichment GO analysis revealed that DEGs were functionally annotated to 10, 20, 79, 55 GO terms in following comparisons: *S. abrotanoides* roots versus *S. yangii* roots*, S. abrotanoides* leaves versus *S. yangii* leaves, *S. yangii* roots versus leaves; and *S. abrotanoides* roots versus leaves, respectively Supplementary Table S4. Annotated transcripts were linked to the GO categories, including molecular functions (MF), biological processes (BP) and cellular components (CC). GO analysis revealed that protein binding was the most enriched term in the MF category, in all comparisons, except for the one done for leaves. In the BP category, DEGs associated with cellular process, cellular metabolic process and metabolic process were highly enriched, again in all comparisons, except leaves. DEG enrichment in the cellular metabolic term suggests that the secondary metabolic pathways in roots and leaves of *S. abrotanoides* and *S. yangii* varied significantly. Among various secondary metabolic pathways, only phenylpropanoid biosynthesis was pointed out by the analysis, showing significantly different transcriptional levels of six genes, including *4-coumaryl-CoA ligase* (*4CL*). The phenylpropanoid pathway in *S. abrotanoides* and *S. yangii* was already analysed by us earlier^25^. The diterpenoid pathway was not enriched in present analysis, which may be explained in two ways. Either variations in the expression of diterpenoid pathway-related genes were not significant, which could be attributed to the similar upstream pathway steps, or the genes related to the downstream steps of diterpenoid biosynthesis pathway were not present in the databases which were used for GO enrichment analysis. There is, however, one gene – *ISPF*, an ortholog of the 2-C-methyl-D-erythritol 2,4-cyclodiphosphate synthase in *S. miltiorrhiza* (*SmMCS*), involved in the plastidic MEP pathway, which is associated with multiple GO terms in MF, BP and CC categories, such as: cellular metabolic process, organic substance metabolic process, phosphorus metabolic process, chlorophyll metabolic process, tetrapyrrole metabolic process as well as various plastid compartments and structures. A high enrichment of this DEG, related to the upstream terpenoid biosynthesis pathway, may point out towards lower steps of the diterpenoid pathway, which could possibly be enriched, if only the genes involved in them were present in the database. In the CC category, the enrichment analysis revealed largely enriched DEGs connected to plastid and chloroplast components, in comparisons performed between roots and leaves of both species, which is consistent with differences in the physiological functions of aerial and underground parts of plants, especially with regards to photosynthesis. Also, two KEGG processes (“Metabolic pathways” and “Photosynthesis”) were enriched in functional annotations Supplementary Table S4. The most interesting GO terms were visualized on Fig. 4b.

### Expression analysis of DEGs involved in abietane diterpenoid pathway

To investigate the abietane diterpenoid metabolism in *S. abrotanoides* and *S. yangii*, a homology analysis using the genome information of *S. miltiorrhiza* and other species of *Salvia* was performed along with the tissue-specific expression analysis of selected candidate unigenes. Further transcriptome mining revealed existence of 39 different transcripts related to abietane diterpenoid biosynthesis in transcriptomes of *S. abrotanoides* and *S. yangii*. Out of them, 9 transcripts were found to be homologous to genes from the cytosolic MVA pathway, 12 were homologs of plastidic MEP genes, one transcript turned out to be a homolog of the isopentenyl diphosphate isomerase (IDI) gene, and 17 transcripts were found to be related to the downstream steps of biosynthesis of abietanoids and nor-abietanoids (Table 2).

**Table 2:** Expression of genes related to tanshinone biosynthesis pathway (FPKM).

Gene name	Gene abbreviation	Homolog NCBI ID	Transcript ID	SA_R1	SA_R2	SY_R1	SY_R2	SA_L1	SA_L2	SY_L1	SY_L2
MVA pathway (cytosol)											
*Acetyl-CoA C-acetyltransferase*	*AACT*	JN831101	DN41649_c0_g1_i4	15.3	16.2	19.6	19.6	18.0	16.4	19.1	19.4
*Acetyl-CoA C-acetyltransferase*	*AACT*	EF635969	DN45648_c2_g1_i13	63.0	59.8	44.0	42.2	44.1	43.8	57.2	54.3
*3-Hydroxy-3-methylglutaryl coenzyme A synthase*	*HMGS*	FJ785326	DN44189_c4_g1_i3	77.5	76.3	41.8	42.9	43.5	45.5	65.6	65.9
*3-Hydroxy-3-methylglutaryl-coenzyme A reductase*	*HMGR*	JN831103	DN46275_c4_g11_i1	9.6	9.3	6.6	6.5	0.2	0.3	0.0	0.0
*3-Hydroxy-3-methylglutaryl-coenzyme A reductase*	*HMGR*	EU680958	DN47604_c3_g3_i2	0.0	0.0	23.5	24.7	0.0	0.0	1.8	1.9
*3-Hydroxy-3-methylglutaryl-coenzyme A reductase*	*HMGR*	JN831102	DN47604_c3_g3_i1	0.0	0.0	511.3	513.4	0.0	0.0	114.0	135.5
*Mevalonate kinase*	*MK*	JN831104	DN45365_c0_g2_i2	33.5	34.6	38.4	38.5	16.5	17.6	17.3	17.9
*5-Phosphomevalonate kinase*	*PMK*	JN831095	DN41885_c0_g1_i1	12.0	11.4	9.5	9.2	9.0	9.3	9.9	10.7
*Mevalonate diphosphate decarboxylase*	*MDC*	JN831105	DN41129_c0_g1_i3	52.2	52.9	41.0	42.6	13.9	14.2	21.1	19.5
MEP pathway (plastid)											
*1-Deoxy-D-xylulose 5-phosphate synthase*	*DXS*	EU670744	DN43908_c1_g2_i7	32.3	35.0	59.3	59.2	194.3	185.8	82.8	85.7
*1-Deoxy-D-xylulose 5-phosphate synthase*	*DXS*	FJ643618	DN43908_c1_g1_i2	201.3	204.6	59.0	61.3	38.2	26.4	7.3	6.0
*1-Deoxy-D-xylulose 5-phosphate synthase*	*DXS*	JN831117	DN45932_c0_g1_i2	14.7	14.3	11.9	11.8	18.5	17.9	13.1	13.2
*1-Deoxy-D-xylulose 5-phosphate synthase*	*DXS*	JN831116	DN43908_c1_g4_i3	1.8	2.1	7.0	6.7	0.6	0.7	0.2	0.3
*1-Deoxy-D-xylulose 5-phosphate synthase*	*DXS*	JN831118	DN43908_c1_g3_i3	0.1	0.5	1.2	1.4	0.7	0.7	3.4	3.4
*1-Deoxy-D-xylulose5-phosphate reductoisomerase*	*DXR*	DQ991431	DN47013_c4_g1_i8	54.4	52.4	30.9	29.5	130.6	119.4	110.5	105.4
*2-C-methyl-D-erythritol 4-phosphate cytidylyltransferase*	*MCT*	JN831096	DN46413_c3_g5_i1	12.9	11.5	9.3	8.9	28.5	30.8	34.4	33.9
*4-diphosphocytidyl-2-C-methyl-D-erythritol kinase*	*CMK*	EF534309	DN47497_c1_g1_i2	47.3	44.1	21.9	22.8	32.3	32.2	28.9	28.3
*2-C-methyl-D-erythritol 2,4-cyclodiphosphate synthase*	*MCS*	JX233816	DN41571_c0_g1_i2	77.2	77.1	38.2	36.5	361.4	352.1	280.1	256.8
*4-Hydroxy-3-methylbut-2-enyl diphosphate synthase*	*HDS*	KJ746807	DN44689_c0_g1_i10	174.9	172.4	100.7	100.0	594.7	575.3	354.9	349.9
*4-Hydroxy-3-methylbut-2-enyl diphosphate synthase*	*HDS*	JN831098	DN44689_c0_g1_i11	0.0	0.0	1.2	1.0	0.0	0.0	0.0	0.1
*4-Hydroxy-3-methylbut-2-enyl diphosphate reductase*	*HDR*	JN831099	DN46931_c0_g1_i3	183.7	186.5	111.9	112.1	1870.1	1887.6	1013.2	943.2
Downstream tanshinone biosynthesis pathway											
*Isopentenyl diphosphate isomerase*	*IDI*	JN831106	DN43304_c4_g2_i1	22.2	22.0	3.3	3.1	23.4	23.7	3.9	4.3
*Isopentenyl pyrophosphate isomerase*	*IPI*	EF635967	DN43742_c3_g2_i4	79.4	81.7	70.5	72.0	34.5	34.5	37.2	39.4
*Farnesyl pyrophosphate synthetase*	*FPPS*	HQ687768	DN41600_c0_g1_i5	74.8	73.3	47.6	46.0	30.1	28.7	43.6	44.0
*Geranyl diphosphate synthase SSUII.2*	*GPPS*	JN831110	DN29144_c0_g2_i1	13.9	14.0	16.0	16.3	74.2	71.5	62.0	58.6
*Geranyl diphosphate synthase SSUII.1*	*GPPS*	JN831109	DN29144_c0_g1_i1	53.6	55.0	28.1	26.7	5.6	3.9	0.8	0.5
*Geranyl diphosphate synthase*	*GPPS*	JN831107	DN47317_c2_g2_i4	13.9	12.9	15.6	14.8	16.1	18.8	19.5	20.7
*Geranyl diphosphate synthase LSU*	*GPPS*	JN831111	DN45200_c2_g5_i1	5.3	5.5	3.8	4.2	4.7	3.5	1.0	0.7
*Geranyl diphosphate synthase SSUI*	*GPPS*	JN831108	DN38777_c0_g2_i1	15.1	14.7	24.7	23.3	6.1	3.7	1.7	1.0
*Geranylgeranyl diphosphate synthase*	*GGPPS*	FJ178784	DN45200_c2_g1_i1	83.5	84.9	20.6	19.5	44.6	43.7	37.8	36.7
*Geranylgeranyl diphosphate synthase*	*GGPPS*	JN831112	DN45200_c2_g3_i2	1.7	1.0	0.4	0.3	0.9	0.8	1.5	1.5
*Geranylgeranyl diphosphate synthase*	*GGPPS*	JN831113	DN45200_c2_g2_i1	4.4	4.0	1.2	1.4	2.0	2.0	1.1	1.1
*Copalyl diphosphate synthase*	*CPS*	EU003997	DN39347_c0_g1_i2	155.8	154.7	55.5	56.6	3.1	3.1	5.9	5.9
*Copalyl diphosphate synthase*	*CPS*	JN831114	DN31732_c0_g1_i1	0.0	0.0	0.0	0.0	4.0	3.0	0.7	0.5
*Copalyl diphosphate synthase*	*CPS*	JN831115	DN80384_c0_g1_i1	0.3	0.3	1.0	1.2	0.0	0.0	0.1	0.1
*Copalyl diphosphate synthase*	*CPS*	JN831121	DN18051_c0_g1_i1	0.4	0.5	2.2	2.1	0.0	0.1	0.6	0.9
*Kaurene synthase*	*KSL1*	EF635966	DN47645_c2_g2_i1	95.1	95.6	28.8	27.1	20.6	18.0	7.3	7.7
*Kaurene synthase*	*KSL2*	JN831119	DN46601_c0_g3_i10	10.4	9.1	9.0	7.9	5.1	5.9	5.6	7.2
*cytochrome P450*	*CYP76AH89*	*CYP76AH23 Rosmarinus officinalis*	DN46294_c1_g1_i3	1290.2	1357.5	543.1	543.4	222.9	155.2	57.3	46.2
*ferruginol synthase/cytochrome P450*	*CYP76AH90*	*CYP76AH4 Rosmarinus officinalis*	DN46294_c1_g1_i4	9.7	9.9	4.5	4.5	8.4	8.2	17.0	20.5
*cytochrome P450*	*CYP76AH91*	*CYP76AH6 Rosmarinus officinalis*	DN102248_c0_g1_i1	6.6	6.8	0.5	0.5	0.8	0.8	2.2	1.8
*cytochrome P450*	*CYP76AK25*	*CYP76AK5v1 Salvia miltiorrhiza*	DN47322_c0_g2_i7	645.5	677.7	231.8	209.9	25.9	17.2	2.7	2.4
*carnosic acid synthase/cytochrome P450*	*CYP76AK-fragment1*	*CYP76AK18 Salvia dorisiana*	DN44093_c0_g1_i2	656.1	728.7	155.1	163.0	37.3	29.6	6.2	3.5
*2-oxoglutarate-dependent dioxygenase/ tanshinone IIA synthase*	*2-ODD14*	*2-ODD14/ TIIAS* *Salvia miltiorrhiza*	DN36849_c0_g1_i1	31.8	34.5	14.8	15.8	22.8	23.4	16.3	13.8
*cytochrome P450*	*CYP71D754*	*CYP71D411 Salvia miltiorrhiza*	DN45603_c2_g5_i2	604.5	620.5	35.2	35.7	2.9	3.0	0.1	0.1
*cytochrome P450*	*CYP71BE213*	*CYP71BE52 Salvia pomifera*	DN45603_c2_g4_i9	105.1	107.4	42.2	41.5	85.0	78.0	32.5	29.1

**Figure 5:**
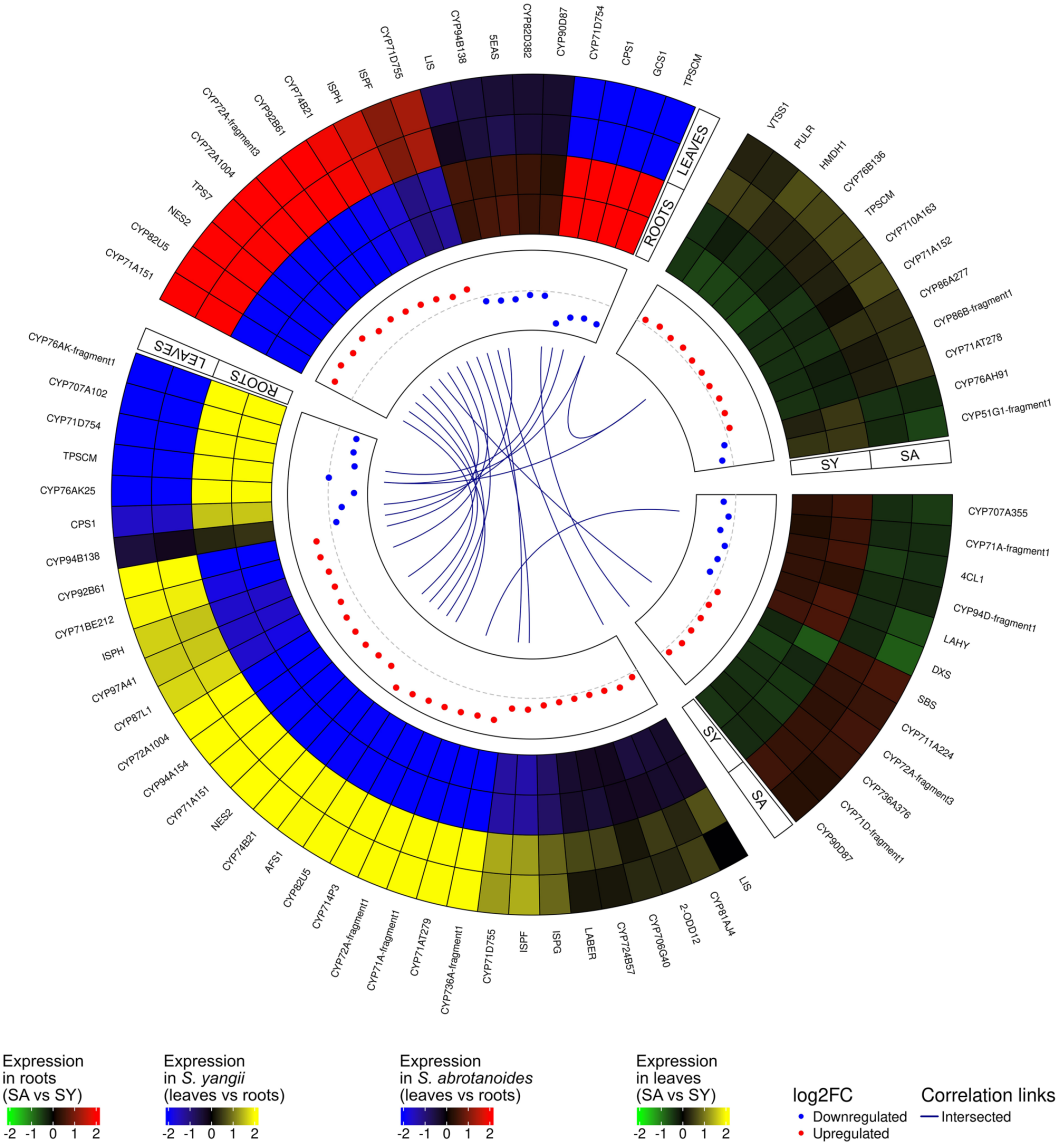
Circular visualization of trans-interactions occurring between differentially expressed protein-coding genes (DEGs) related to diterpenoid metabolism and other candidate genes revealed by transcriptome mining of roots and leaves of *S. abrotanoides* and *S. yangii*. Heatmaps show the expression profiles of DEGs in roots (green–red) and leaves (green–yellow) of both species as well as DEGs in *S. yangii* (blue–yellow) and *S. abrotanoides* (blue–red). DEGs were arranged according to increasing log2(fold change). Details of the interactions are provided in the text. Gene abbreviations are explained in the text and in the Supplementary Tables S3, S5 and S6.

In transcriptomes of *S. abrotanoides* and *S. yangii*, several key enzymes, such as AACT, HMGR, DXS, HDS, GGPPS, CPS, and KSL are present as multiple transcripts with different sequence similarity to known genes and with different expression patterns in analysed plant organs (Table 2). According to the current understanding of the abietane diterpenoid biosynthesis, cytochrome P450s (CYPs) play a key role in the downstream steps of the pathway. 44 CYPs were identified among DEGs in this study (Supplementary Table S5) and their annotation was kindly provided by Prof. David Nelson^59^. Out of them, seven are thought to be related to the abietane diterpenoid biosynthesis (Table 2). A total of 16 transcripts homologous to the 2-ODDs of *S. miltiorrhiza* were detected in transcriptomes of *S. abrotanoides* and *S. yangi* (Supplementary Table S6), out of which one was found to share the highest sequence similarity with the Sm2-ODD14 designated as the tanshinone IIA synthase (TIIAS)^33^ and therefore was included into further analysis (Table 2). Many of the above-mentioned genes were found among DEGs detected in the study. The trans-interactions occurring between DEGs related to diterpenoid metabolism and other candidate genes revealed by transcriptome mining of roots and leaves of *S. abrotanoides* and *S. yangii* along with their expression profile are presented on Fig. 5. This subset of candidate DEGs has been arranged according to increasing log2(fold change) and presented as heatmaps. The analysis of trans-interactions between selected DEGs revealed that genes expressed differentially in roots and leaves of *S. abrotanoides* (blue–red), such as CPS1 or CYP71D754, appear also as DEGs in *S. yangii* (blue–yellow) and are found to be corelated as they share similar expression pattern. In contrast, candidate genes differentially expressed in roots of *S. abrotanoides* and *S. yangii* (green–red) and candidate genes expressed differentially in leaves of both species (green–yellow) represent different subsets which are found not to be correlated with each other. Only one root-DEG, CYP71A-fragment1, homologous to *S. miltiorrhiza* CYP71A57, and one leaf-DEG – TPSCM, homologous to *S. splendens* gamma-cadinene synthase, were shown to be among *S. yangii* DEGs.

**Figure 6:**
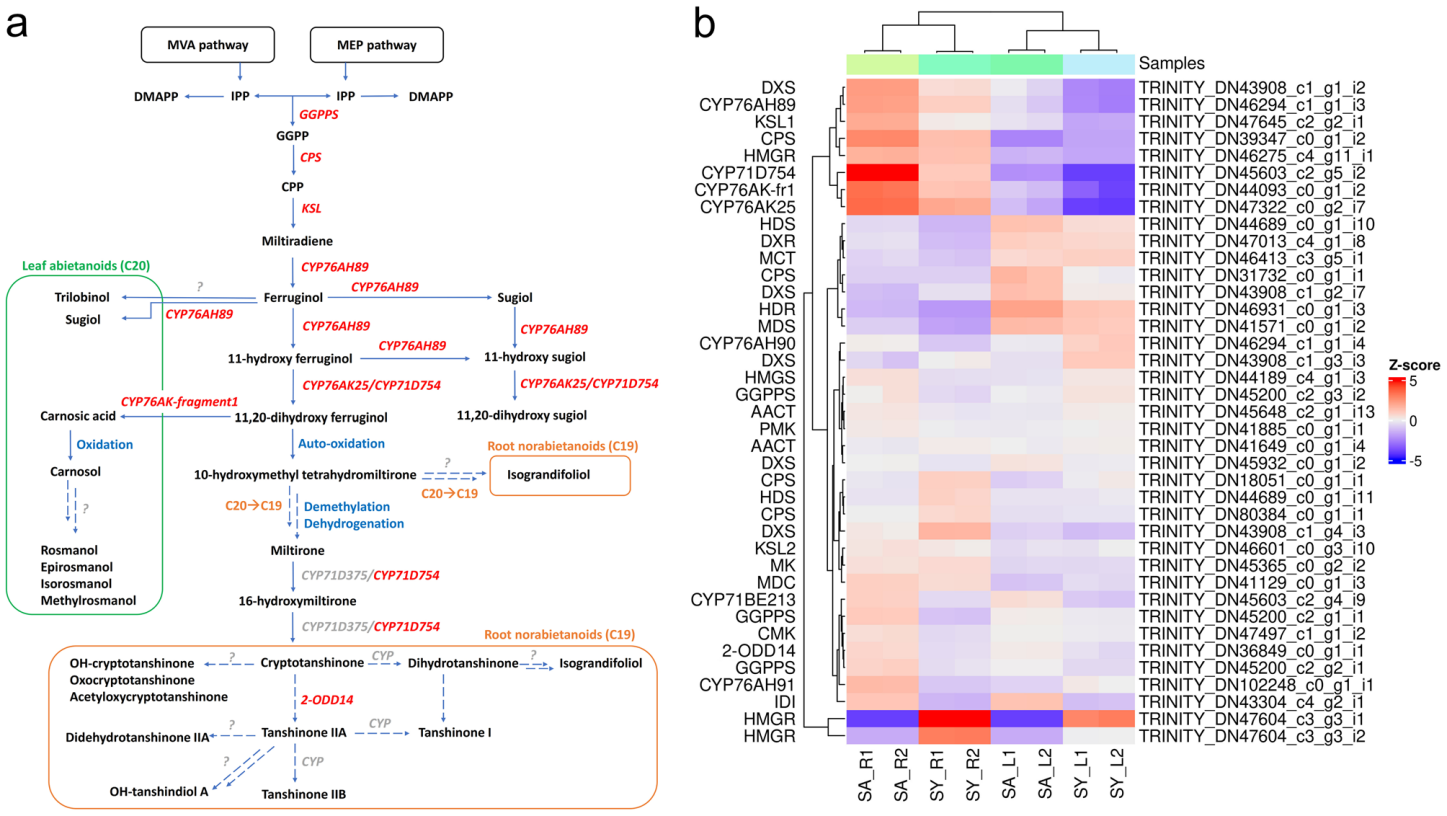
A proposed biosynthetic pathway of abietane-type diterpenoids in *S. abrotanoides* and *S. yangii*. (**a**) Pathways and genes involved in the biosynthesis of abietane diterpenoids. (**b**) Gene expression heatmap (Z-score of the FPKM value) of the identified candidate genes in roots and leaves of *S. abrotanoides* and *S. yangii*, clustered according to their expression profile. Gene abbreviations are explained in the text and in the Table 2.

Based on the elucidated abietane diterpenoid biosynthesis pathway in other *Salvia* plants, including *S. miltiorrhiza*, *S. fruticosa*, and *S. officinalis*^26,49,50,57,58^, we predicted the analogous pathway possibly operating in *S. abrotanoides* and *S. yangii* (Fig. 6a). Among 39 transcripts related to abietane diterpenoid biosynthesis in transcriptomes of *S. abrotanoides* and *S. yangii*, eight formed a cluster of root-specific genes, when clustered according to their expression profile (Fig. 6b and Table 2). Although many of the MEP and MVA pathway enzymes were found to be present as multiple transcripts in *S. abrotanoides* and *S. yangii*, two root-specific transcripts were detected: DXS (TRINITY_DN43908_c1_g1_i2) and HMGR (TRINITY_DN46275_c4_g11_i1). Interestingly, two HMGR isoforms (TRINITY_DN47604_c3_g3_i1 and TRINITY_DN47604_c3_g3_i2) have been detected in roots and leaves of *S. yangii* and not in *S. abrotanoides*. Among the enzymes responsible for general diterpenoids catalytic steps leading to the biosynthesis of miltiradiene, a root-specific isoforms of CPS (TRINITY_DN39347_c0_g1_i2) and KSL (TRINITY_DN47645_c2_g2_i1) were found and selected for further analysis.

A thorough transcriptome mining has been performed in this work in order to search for sequences homologous to the SmCYP76AH1, a crucial enzyme for the biosynthesis of tanshinones in *S. miltiorrhiza*^49,51^. Surprisingly, none of the CYPs in *S. abrotanoides* and *S. yangii* was found to share the similarity high enough to be designated as an ortholog of SmCYP76AH1.

The search for sequences homologous to the SmCYP76AH3^52^ resulted in the discovery of three cytochromes from the CYP76AH clad, in transcriptomes of *S. abrotanoides* and *S. yangii* (Supplementary Table S5 and Fig. 6). CYP76AH89, represented by the transcript isoform TRINITY_DN46294_c1_g1_i3, shares the highest similarity (93.3%) with the CYP76AH23 from *S. rosmarinus*, the similarity with the CYP76AH22 from *S. rosmarinus*, CYP76AH24 from *S. fruticosa* and CYP76AH3 from *S. miltiorrhiza* also exceeded 90%. Although the TRINITY_DN46294_c1_g1_i4 transcript was initially regarded by the bioinformatic analysis as an isoform of the CYP76AH89 gene, due to 85% mutual sequence similarity, detailed analysis of the translated protein sequence of both isoforms and their homologs convinced us to designate the TRINITY_DN46294_c1_g1_i4 as a possibly separate gene – CYP76AH90. It shares 89.1% of identity with CYP76AH4 from *S. rosmarinus*, which is capable of hydroxylating the abietatriene^50^, as well as 87% of identity with the SmCYP76AH1. The third CYP of the CYP76AH clade is represented by the TRINITY_DN102248_c0_g1_i1 transcript showing the highest similarity (92.2%) with CYP76AH6 from *S. rosmarinus*, and was named CYP76AH91. 89.5% of nucleotide identity was also found between the CYP76AH91 sequence and ferruginol synthase-like from both *S. splendens* and *S. hispanica*. Detailed analysis of translated protein sequences of CYP76AHs from *S. abrotanoides* and *S. yangii* and their homologs are presented in the next paragraph. Expression analysis of three CYP76AHs detected in this work clearly show that the CYP76AH89 is mainly involved in the tanshinone biosynthesis. The expression level of CYP76AH89 reached over 543 FPKM in roots of *S. yangii* and 1290.2 FPKM and 1357.5 FPKM in two transcriptomic library replicas of *S. abrotanoides* roots, which is the highest result among diterpenoid-related gene expression and designates the CYP76AH89 a marker gene for the tanshinone biosynthesis in analyzed species (Table 2). The expression level of CYP76AH90 and CYP76AH91 is much lower, reaching 17 and 20.5 FPKM in leaves of *S. yangii* and 6.6 and 6.8 FPKM in roots of *S. abrotanoides*, respectively (Table 2).

TRINITY_DN47322_c0_g2_i13 transcript (named CYP76AK25) shares 83.8% of sequence identity with the SmCYP76AK5 of unknown function (Supplementary Table S5x and Fig. 6). This transcript is also similar in 73.8% and 72.5% to the SmCYP76AK3 and 11-hydroxysugiol 20-monooxygenase-like from *S. splendens*, respectively. The expression of CYP76AK25 is root specific, scoring 645.5, 677.7, 231.8 and 209.9 FPKM in two transcriptomic library replicas of roots of *S. abrotanoides* and *S. yangii*, respectively (Table 2).

In *S. abrotanoides* and *S. yangii*, no orthologs of the SmCYP71D373 and SmCYP71D375 were detected. However, the TRINITY_DN45603_c2_g5_i2 transcript (named CYP71D754) was found to be similar in 86.8% to SmCYP71D411, which is known to catalyze the hydroxylation at C20 (of sugiol) but is not capable to perform the heterocyclization reaction (Supplementary Table S5 and Fig. 6). The analysis of the translated protein sequence of both the CYP71D754 and SmCYP71D411 revealed that both cytochromes have identical active sites residues required for the enzymatic reaction with miltirone^53^. The expression of CYP71D754 turned out to be root-specific, reaching 604.5 FPKM and 620.5 FPKM in *S. abrotanoides* roots and 35.2 FPKM and 35.7 FPKM in the roots of *S. yangii* (Table 2).

Out of 16 transcripts homologous to the Sm2-ODDs detected in transcriptomes of *S. abrotanoides* and *S. yangii*, TRINITY_DN36849_c0_g1_i1 (2-ODD14) was found to share 84.7% of sequence similarity with the Sm2-ODD14^33^ (Supplementary Table S6). Its expression, however, was slightly higher in roots of *S. abrotanoides* (31.8 FPKM and 34.5 FPKM) than in leaves or at the same level in both organs of *S. yangii* (Table 2). Other isoforms with more apparent root-specific expression have been found in transcriptomes of *S. abrotanoides* and *S. yangii*, which makes them interesting candidates to be tested towards tanshinone biosynthesis.

Two other cytochromes P450s, which could be related to abietane diterpenoids biosynthesis, have been found through transcription mining. A TRINITY_DN44093_c0_g1_i2 partial transcript, which translates into the 212 amino acid-long fragment possesses 86.3% similarity to CYP76AK18 from *S. dorisiana*, and has been named CYP76AK-fragment1 (Supplementary Table S5). Additional BLAST analysis showed that this partial CYP sequence shares 90% similarity with the carnosic acid synthase CYP76AK8 from *S. rosmarinus*. RoCYP76AK6-8 have been demonstrated to catalyze three sequential C20 oxidations for the conversion of 11-hydroxyferruginol to carnosic acid in yeast^58^. Due to its high expression in roots of *S. abrotanoides* (656.1 and 728.7 FPKM) and *S. yangii* (155.1 and 163 FPKM), CYP76AK-fragment1 clusters together with other root-specific genes found by our analysis (Fig. 6b and Table 2). So, sequence similarity analysis of CYP76AK-fragment1 strongly suggests its involvement in the biosynthesis of carnosic acid in *S. abrotanoides* and *S. yangii*, however, its expression profile obtained by RNA-seq does not confirm this hypothesis.

TRINITY_DN45603_c2_g4_i9 transcript (CYP71BE213) was found to be similar in 77.84% to the CYP71BE52 from *S. pomifera*, which is highly expressed in its glandular trichomes and oxidizes ferruginol at position 2α to produce salviol^43,57^ (Supplementary Table S5).

### Nucleotide sequence analysis of *CYP76AHs*

**Figure 7:**
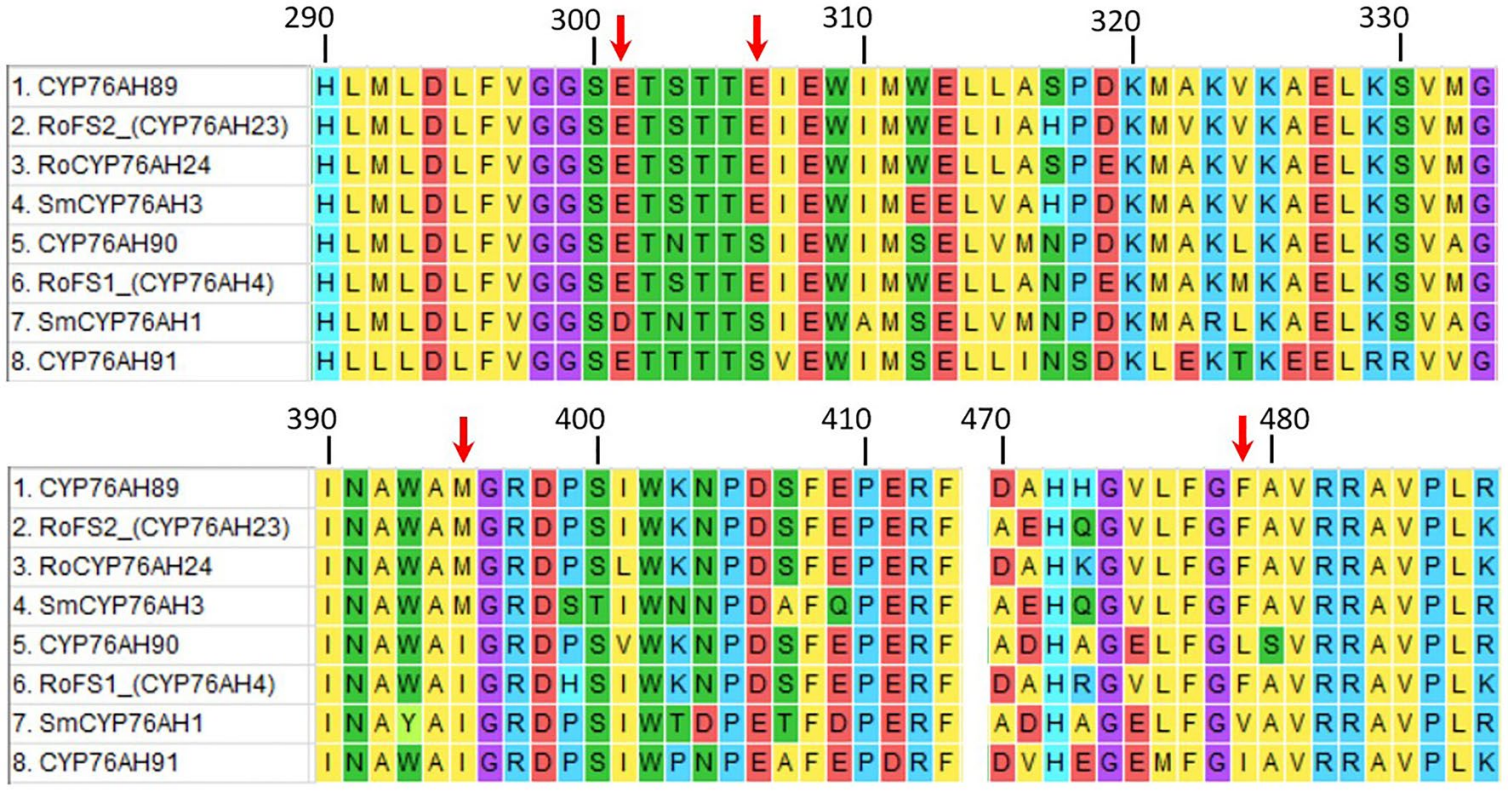
Alignment of regions around the four distinguishing active sites residues of CYP76AH89, CYP76AH90 and CYP76AH91 together with sequences of their homologs. Different colors refer to different amino acids, red arrows point to amino acids of 301, 306, 395 and 479, respectively.

Two representative transcripts isoforms of the gene TRINITY_DN46294_c1_g1 were detected to have notably different nucleotide sequence which, despite sharing mutual 85% similarity, translated into significantly different protein sequences. Partial sequences around the four distinguishing active sites residues of *CYP76AH89* (TRINITY_DN46294_c1_g1_i3) and *CYP76AH90* (TRINITY_DN46294_c1_g1_i4) were amplified and Sanger sequenced (GeneBank at accession numbers: ON365538, ON365539, ON365540, ON365541). A translated protein alignment of resulting sequences together with protein sequences of their homologs and the translated protein sequence of CYP76AH91 is presented in Fig. 7. It has been shown before that four amino acid sites are responsible for the catalytic activity of CYP76HAs, namely: 301, 306, 395 and 479 amino acid residues^60^. The sequencing with Sanger method has confirmed results obtained with the NGS sequencing and demonstrated that the active site of CYP76AH89 consisted of E301, E306, M395, F479, while the CYP76AH90 had E301, S306, I395, L479 in its active site.

### Seasonal variations in expression levels of genes related to biosynthesis of abietane diterpenoids (qRT-PCR)

**Figure 8:**
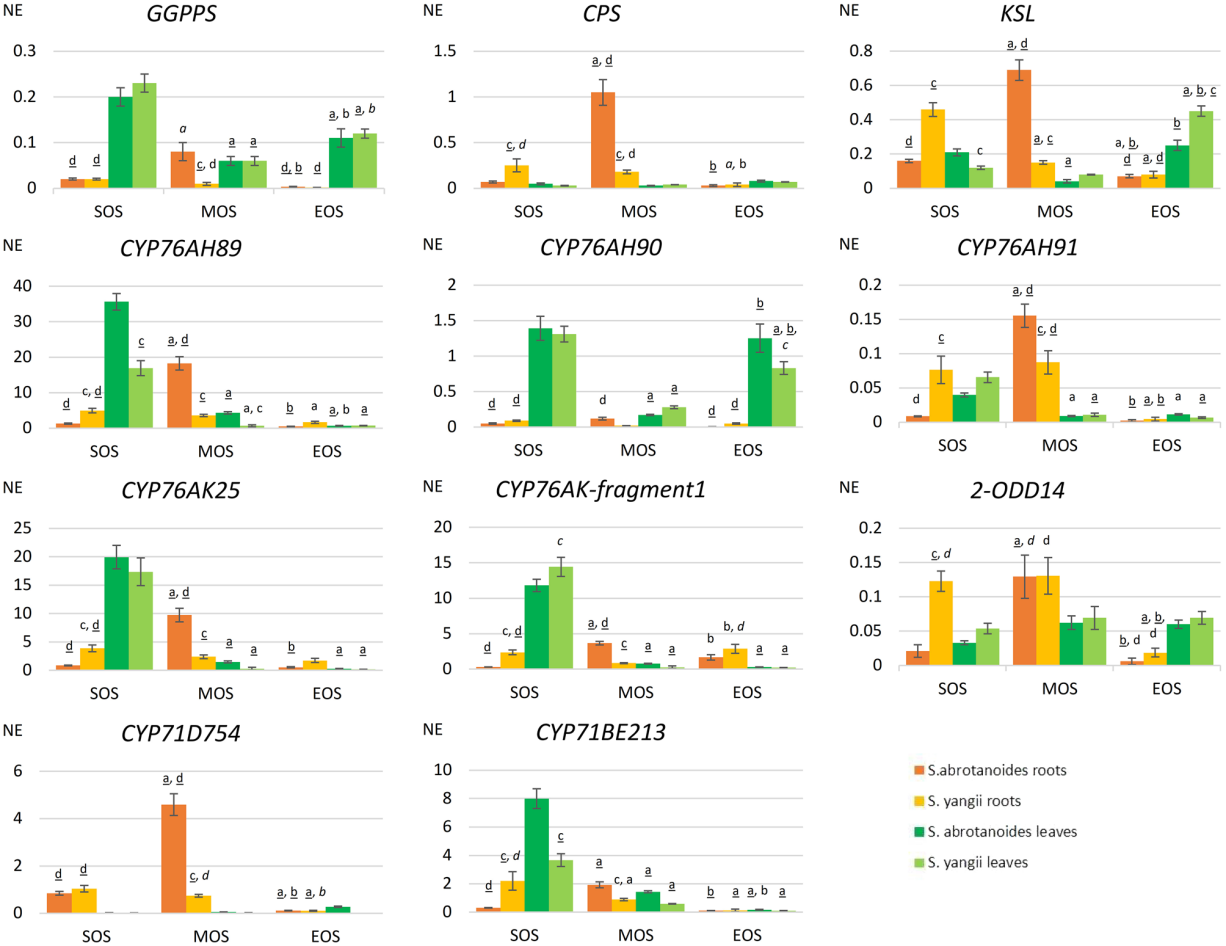
Expression levels of genes related to the downstream biosynthesis pathway of abietane diterpenoids in leaves and roots of *S. abrotanoides* and *S. yangii* in three growth stages during the vegetative season (SOS – start of season, MOS – middle of season, EOS – end of season). Bars represent transcript levels normalized to *ACT*. NE, normalized expression. The letters above the error bars refer to statistically significant differences as follows: a – significant differences in comparison to SOS, b – significant differences in comparison to MOS, c – significant differences in comparison to *S. abrotanoides*, d – significant differences in comparison to leaves. The level of significance is indicated by the used fonts: p < 0.01 lower case font, p < 0.001 italic font, p < 0.0001 underlined font.

As described above, we discovered novel candidate genes possibly involved in the biosynthesis of abietane-type diterpenoids in *S. abrotanoides* and *S. yangii*. Eleven genes from the downstream biosynthesis pathway were selected for extended analysis of their expression during the vegetation season. Quantitative real-time PCR analysis was used to investigate the expression pattern of *GGPPS* (TRINITY_DN45200_c2_g1_i1), *CPS* (TRINITY_DN39347_c0_g1_i2), *KSL* (TRINITY_DN47645_c2_g2_i1), *CYP76AH89* (TRINITY_DN46294_c1_g1_i3), *CYP76AH90* (TRINITY_DN46294_c1_g1_i4), *CYP76AH91* (TRINITY_DN102248_c0_g1_i1), *CYP76AK25* (TRINITY_DN47322_c0_g2_i7), *CYP76AK-fragment1* (TRINITY_DN44093_c0_g1_i2), *2-ODD14* (TRINITY_DN36849_c0_g1_i1), *CYP71D754* (TRINITY_DN45603_c2_g5_i2), and *CYP71BE213* (TRINITY_DN45603_c2_g4_i9) in leaves and roots of *S. abrotanoides* and *S. yangii*. Gene transcript levels in each sample were normalized to the respective transcript level of *ACT*, which generated a normalized expression value (NE) (Fig. 8 and Supplementary Table S7). The expression of selected genes was analysed with the quantitative real-time PCR in three time points: at the start of the season (SOS), in the middle of the season (MOS) and at the end of the season (EOS), so the expression level at MOS corresponded with the amount of transcript measured by NGS.

The analysis revealed relatively low expression of genes encoding for general diterpenoids enzymes. The highest expression of *GGPPS* was detected in leaves of *S. abrotanoides* and *S. yangii* at the start of the season. The highest transcript levels of *CPS* and *KSL* were obtained in roots of *S. abrotanoides* in the middle of season, which corresponded with the RNA-seq results. In roots of *S. yangii* the transcript levels of *CPS* and *KSL* were significantly lower at MOS and significantly higher in SOS.

The highest expression of all analysed genes was obtained for the *CYP76AH89*. In roots, its transcript levels were the highest at MOS and reached 18.21 and 3.61 in *S. abrotanoides* and *S. yangii*, respectively, which is in line with levels obtained by RNA-seq. In leaves, the peak of expression was noted at the start of the season, reaching 35.62 (*S. abrotanoides*) and 16.87 (*S. yangii*). The very high expression of *CYP76AH89* in leaves and roots suggested its involvement in the biosynthesis of both tanshinones and carnosic acid, with notably different spatial and temporal expression pattern though. The expression levels of *CYP76AH90 and CYP76AH91* were much lower than those of the *CYP76AH89*, proving their minor involvement in the abietane biosynthesis. The *CYP76AH90* exhibited relatively high expression in leaves of both species (at SOS and EOS), while the *CYP76AH91* had a highest expression in roots of *S. abrotanoides* and *S. yangii* at MOS. Significantly different transcript levels and expression patterns of the three detected CYP76AHs proved the hypothesis that all three enzyme isoforms operate in *S. abrotanoides* and *S. yangii*, with the *CYP76AH89* playing the major role in the abietane biosynthesis.

Similarly to *CYP76AHs*, both *CYP76AKs* genes showed the highest expression levels in leaves, at the start of the season. *CYP76AK25* leaf transcript levels reached at SOS 19.92 and 17.32, while *CYP76AK-fragment1* reached 11.81 and 14.42 in *S. abrotanoides* and *S. yangii*, respectively. In roots the expression pattern of *CYP76AKs* varied between species, but an involvement of both cytochromes in biosynthesis of tanshinones and carnosic acid was implied by their expression. The transcript levels of *CYP76AKs* at MOS corresponded with those obtained by RNA-seq.

*2-ODD14* expression was shown to be at a relatively low level in both organs and species. In leaves, *2-ODD14* was quite stably expressed over the season, in roots significantly higher transcript levels were recorded in MOS for both *S. abrotanoides* and *S. yangii*, and at SOS in *S. yangii*.

*CYP71D754* appeared to be a root-specific gene with the expression hardly detectable in leaves, in all three analysed time points. Its expression in roots begun already at the start of the season, increased significantly in *S. abrotanoides* in MOS (4.59) while in *S. yangii* it was kept at the similar level and dropped to near-zero levels at the end of the season in both species. Again, the transcripts levels detected by qRT-PCR at MOS were in accordance with those revealed by NGS.

The *CYP71BE213* exhibited the highest expression at the start of the season in leaves and roots of *S. yangii*. At MOS, the expression level in *S. abrotanoides* roots increased, while the other dropped down. Comparable, but slightly higher in roots, transcript levels were recorded by both methods, qRT-PCR and RNA-seq.

## Discussion

Abietane diterpenoids, including tanshinones and carnosic acid, are the most abundant type of tricyclic terpenoids, the biosynthetic pathway of which has been best investigated^45,61,62^. Tanshinones, carnosic acid and its derivatives are present in several species of *Lamiaceae*, in various combinations. It is known that the family of CYP450s plays an essential role in decoration of diterpene skeletons, providing for various types of structural oxidative modification reaction, such as: hydroxylation, carbonylation and heterocyclization^48,49,52,53,57^. Additionally, it has been shown that the catalytic promiscuity of CYP450s creates a metabolic grid for tanshinone biosynthesis^52,63^. Nonetheless, despite numerous experimental strategies to elucidate the tanshinone biosynthesis pathway has been implemented to date, including: investigation of a spatial organization of the diterpenoid biosynthetic gene cluster^64^, comparative metabolomic, transcriptomics and proteomic analysis^33,40,44,65^, targeted mutagenesis approaches using RNA interference and CRISPR/Cas9 system^41,66,67^, hairy roots cultures and elicitor treatments^31,68–74^, or endophytic fungi infection^75^, several steps from the pathway remain unresolved. Particularly, the most intriguing transformation – the hypothetical conversion of 11,20-dihydroxyferruginol to miltirone, which requires loss of C20 (directly via demethylation or via decarboxylation of carnosic acid as an intermediate) and aromatization of the “B” ring, has not been determined yet. Also, downstream transformations involving modification at the C4 of the miltirone backbone and leading to the formation of the metabolic grid of tanshinones remain undetermined as well as the direct precursor and transformations leading to the biosynthesis of isograndifoliol^76^ and trilobinol^77^.

Utilising two closely related species of slightly distinct diterpenoid profile, the metabolomics-guided transcriptomic approach allowed to select candidate genes, which expression might have led to observed chemical differences. Firstly, we have used the UHPLC-QTOF-MS analysis to follow the content of diterpenoids in the course of vegetation season (Table 1). Eight compounds were estimated quantitatively, out of which carnosol was the most abundant compound in the leaves of both species while in roots, the profile of abietane diterpenoid compounds was clearly dominated by cryptotanshinone (**11**). Interestingly, several compounds were found to be unique for one or the other species. Sugiol (**9**) was detected only in the *S. yangii* leaves, while trilobinol (**10**) was unique for *S. abrotanoides*. Didehydrotanshinone IIA (**18**), acetyloxycryptotanshinone (**14**) and ketoisograndifoliol (**16**) were unique for the roots of *S. yangii*. Also, the levels of isograndifoliol (**15**) and OH-tanshindiol A (**17**) were significantly higher in the roots of *S. yangii* than in *S. abrotanoides*. This analysis has confirmed our previous observations of a diverse diterpenoid profiles of *S. abrotanoides* and *S. yangii*, although it became clear that the difference in the content of isograndifoliol is quantitative, not qualitative, as previously thought^28^. Current observations corroborate with those made earlier in the roots of *S. yangii*, where the profile of abietane diterpenoid compounds was also dominated by the cryptotanshinone, followed by 1,2-didehydrotanshinone, miltirone and low amounts of tanshinone IIA^78^. Although the presence of tanshinone IIA was detected in roots of both *S. abrotanoides* and *S. yangii*^28^, its low amounts did not allow to analyse its content quantitatively in this work.

As metabolic differences are reflected in different transcript profile of a species or tissues, we performed comparative analysis of *S. abrotanoides* and *S. yangii* transcriptomes. Using an RNA-sequencing technology we have sequenced and de novo assembled transcriptomes of leaves and roots of *S. abrotanoides* and *S. yangii*. As a result, 134,443 transcripts were annotated by UniProt and 56,693 of them were assigned as Viridiplantae. To our knowledge, this is first transcriptomic data reported for these species. In order to seek for differences between sequenced transcriptomes, the differential expression analysis was performed, which revealed multiple genes indicating changes in expression in four comparisons made between *S. abrotanoides* and *S. yangii* leaves and roots (Fig. 3a). GO enrichment analysis of selected DEGs has found many DEGs to be enriched in the cellular metabolic term, which suggested significant variations in the secondary metabolic pathways in roots and leaves of these species. Additionally, related to the upstream terpenoid biosynthesis pathway, the *ISPF* gene (orthologous to the *SmMCS*), was found to be highly enriched in multiple GO terms, which points out towards lower steps of the diterpenoid pathway, currently not present in the databases. The homology and expression of two gene families associated with downstream steps of tanshinone and carnosic acid biosynthesis were studied, namely: cytochromes P-450 and 2-oxoglutarate-dependend dioxygenases. 44 *CYP450s* were identified among DEGs in this study (Supplementary Table S5), out of which seven are thought to be related to the abietane diterpenoid biosynthesis (Table 2). A total of 16 transcripts homologous to the *2-ODDs* of *S. miltiorrhiza* were detected in transcriptomes of *S. abrotanoides* and *S. yangi* (Supplementary Table S6) with one being homologous to the *Sm2-ODD14* designated as the tanshinone IIA synthase (Table 2). Additional BLAST analysis revealed existence of 39 different transcripts related to abietane diterpenoid biosynthesis in transcriptomes of *S. abrotanoides* and *S. yangii*, out of which 17 transcripts were found to be related to the downstream steps of biosynthesis of abietanoids and nor-abietanoids (Table 2) and eight formed a cluster of root-specific genes, when clustered according to their expression profile (Fig. 6b). Finally, eleven candidate genes from the downstream biosynthesis pathway were selected for extended analysis of their expression during the vegetation season.

In transcriptomes of *S. abrotanoides* and *S. yangii*, we have detected three cytochromes from the CYP76AH clade, namely: *CYP76AH89*, *CYP76AH90* and *CYP76AH91* (Supplementary Table S5 and Fig. 6). RNA-seq showed very high transcript levels of *CYP76AH89* in root transcriptomes (at MOS), which was confirmed by qRT-PCR analysis (Table 2 and Fig. 8). Additionally, qRT-PCR analysis revealed a very high expression of *CYP76AH89* in leaf transcriptomes, at the start of the season, which suggests the *CYP76AH89* being a key enzyme in the biosynthesis of both tanshinones and carnosic acid, with notably different expression pattern in leaves and roots. Homology analysis showed that *CYP76AH89* exhibits the highest similarity with the *CYP76AH22* and *CYP76AH23* from *S. rosmarinus* as well as with the *SmCYP76AH3*. In fact, all these enzymes possess the same amino acid residues in their active sites, which are: E301, E306, M395, F479 (Fig. 7). The other two genes from the CYP76AH clade, *CYP76AH90* and *CYP76AH91*, also showed high similarity with ferruginol synthases-like genes from various *Salvia* species, however, their expression levels were much lower than those of the *CYP76AH89*, proving their minor involvement in the abietane biosynthesis. Interestingly, no obvious and significantly expressed homolog of the *SmCYP76AH1* has been found in transcriptomes of *S. abrotanoides* and *S. yangii*. Therefore, we postulate, that the CYP76AH89 may accept both miltiradiene and ferruginol as substrates and catalyses both C11 and C12 hydroxylation, similarly as it has been proved for CYP76AH22-24 from *S. rosmarinus*^26,58^, as well C7 oxidation of the miltiradiene backbone, catalyzed by SmCYP76AH3. The close phylogenetic relationship of *S. abrotanoides* and *S. yangii* with *S. rosmarinus* was demonstrated by us earlier^28^. On the other hand, it was demonstrated that, although CYP76AH1 and CYP76AH3 catalyze two consecutive reactions *in planta*^49,52^, in vitro studies proved them having the same catalytic functions but different catalytic efficiencies. It was found that when ferruginol, 11-hydroxy ferruginol, and sugiol were used as substrates for in vitro enzymatic reactions, CYP76AH1 also catalyzed the hydroxylation of C11 and oxygenation of C7 sites, though at very low catalytic efficiency. Similarly, CYP76AH3 also catalyzed the production of trace amounts of ferruginol from miltiradiene. In the same work, a series of modeling-based mutational variants of CYP76AH1 were designed to integrate the functions of CYP76AH1 and CYP76AH3. The mutant CYP76AH1^D301E,V479F^, which integrated the functions of CYP76AH1 and CYP76AH3, was found to efficiently catalyze C11 and C12 hydroxylation and C7 oxidation of miltiradiene substrates^60^. We hypothesize, that in *S. abrotanoides* and *S. yangii*, the CYP76AH89 might have similarly integrated the two catalytic activities of CYP76AH1 and CYP76AH3 and became a multifunctional enzyme that catalyzes the formation of ferruginol from miltiradiene and further oxidizes the C7 and C11 positions of ferruginol to form sugiol, 11-hydroxy ferruginol, and 11-hydroxy sugiol. The other two CYP76AHs transcripts detected in *S. abrotanoides* and *S. yangii*, *CYP76AH90* and *CYP76AH91*, possessed following predicted amino acid residues in their active site: E301, S306, I395, L479 and E301, S306, I395, I479, respectively (Fig. 7). Whether they possess redundant functions to the CYP76AH89 or contribute to the abundance of different abietane-diterpenoids in *S. abrotanoides* and *S. yangii*, it would need involvement of functional in vitro studies, however, the relatively low expression of *CYP76AH90* and *CYP76AH91* throughout the season (Table 2 and Fig. 8) suggests they minor role in the general biosynthesis pathway of abietanoids.

Next genes possibly encoding for abietane diterpenoid pathway enzymes has been found to be represented by following homologs: *CYP76AK25*, *CYP76AK-fragment1*, *CYP71D754*, *2-ODD14* and *CYP71BE213*.

Both *CYP76AKs* genes cluster together with other root-specific genes found by our RNA-seq analysis (Fig. 6b and Table 2), but the qRT-PCR has additionally revealed their high expression levels in leaves, at the start of the season (Fig. 8). Again, this would suggest involvement of *CYP76AK25* and *CYP76AK-fragment1* in the biosynthesis of both tanshinones and carnosic acid, in, however, different temporal and spatial manner. Homology analysis indicate that CYP76AK25 might catalyze biosynthesis of 11,20-dihydroxysugiol and 11,20-dihydroxyferruginol, while CYP76AK-fragment1 possibly catalyzes three sequential C20 oxidations for the conversion of 11-hydroxyferruginol to carnosic acid in *S. abrotanoides* and *S. yangii* (Fig. 6a) as it was demonstrated for RoCYP76AK6-8^58^.

Given the high similarity of *CYP71D754* with *SmCYP71D411* and its very high expression in roots of *S. abrotanoides* and *S. yangii*, we hypothesize that CYP71D754 may possibly accept the 11-dihydroxysugiol and 11-dihydroxyferruginol as substrates and produce the 11,20-dihydroxysugiol and 11,20-dihydroxyferruginol in these species (Table 2 and Fig. 6). That would make its catalytic activity redundant with the CYP76AK25, however, cytochrome P450s playing redundant roles in plants have already been described^26,63,79^. Whether the CYP71D754 is also able to catalyze the heterocyclization of the D-ring in *S. abrotanoides* and *S. yangii* to produce the cryptotanshinone, remains unknown. On the other hand, given a high level of cryptotanshinone in roots of *S. abrotanoides* and *S. yangii*, an enzyme catalyzing the direct upstream hydroxylation of C16 and 14,16-ether (hetero)cyclization to form the D‐ring (as *SmCYP71D375*) should have a clear root-specific expression profile and in that perspective the *CYP71D754* seems to be a good candidate.

*2-ODD14* was found to share high similarity with the Sm2-ODD14 and had its expression level only slightly higher in roots than in leaves (Table 2 and Fig. 8). Investigation of other isoforms with more apparent root-specific expression would certainly be beneficial, although, the hardly detectable amount of tanshinone IIA in roots of *S. abrotanoides* and *S. yangii* does not allow for expecting a high and root-specific transcript levels of the tanshinone IIA-producing enzyme.

The role of the *CYP71BE213* in the biosynthesis of abietane diterpenoids in *S. abrotanoides* and *S. yangii* could only be speculated, but its homology with salviol-producing CYP71BE52 from *S. pomifera* together with its relatively high expression in roots and leaves of *S. abrotanoides* and *S. yangii* (Table 2 and Fig. 8) and the absence of salviol in the metabolic profile of either of the studied species (Table 1) suggest potential involvement of *CYP71BE213* in other abietane diterpenoid transformations, possibly through the substrate promiscuity of this homolog^43,57^.

Up to date, the commercial supply of tanshinones relies on extraction from *S. miltiorrhiza*. Originally harvested from the wild, *S. miltiorrhiza* is now field cultivated, which has become the main source of Danshen. With good agricultural practices the drug is relatively uniform in quality, but can contain heavy metals and pesticide residues^45^. Therefore, there is a high demand for searching for alternative sources of tanshinones. One of them could be hairy root and cell cultures of *S. miltiorrhiza*, which produce and secrete tanshinones, but the yields from such cultures also does not yet appear to be economically viable^31^. Also, endophytic fungi strains isolated from native *S. abrotanoides* have been reported to be able for *ex planta* biosynthesis of cryptotanshinone^80^, while cultured endophytic fungi from *S. miltiorrhiza* turned out to be capable of tanshinone I and tanshinone IIA production^81,82^. Unfortunately, due to the unknown reasons, endophytic fungi cultures often tend to lose their ability for plant-derived metabolites production^83^. In this view, another tanshinone-producing and easy to grow plant species would serve as a valuable alternative.

Therefore, by using the comparative transcriptomic approach, we have generated a dataset of candidate genes which provides a valuable resource for further elucidation of tanshinone biosynthesis. In a long run, our investigation may lead to optimization of diterpenoid profile in *S. abrotanoides* and *S. yangii* through genetic engineering, which may become an alternative source of tanshinones for further research on their bioactivity and pharmacological therapy.

## Methods

### Plant material

*S. abrotanoides* and *S. yangii* plants were grown in the experimental field of the Botanical Garden of Medicinal Plants (BGMP) at the Wroclaw Medical University (Poland) as previously described^28^. The experimental plants were obtained from the certified collection of the BGMP operating according to the respective bylaws of the Act of Nature Protection of the Republic of Poland and the Ministry of Environment permit (with compliance to the national and global conservation policies, including IUCN Policy Statement on Research Involving Species at Risk of Extinction and the CITES, Convention on the Trade in Endangered Species of Wild Fauna and Flora). Both studied species belong to the non-threatened category for their wide distribution and common occurrence in their natural range.

In the present experiments, we used the roots and leaves of the wild-type clones for which the voucher specimens were deposited in the BGMP’s herbarium under the reference numbers: *S. abrotanoides*: P-122; *S. yangii*: P-123.

The harvest of leaves and roots was performed in mid-May, mid-August and mid-October 2016, which represented three growth stages during the vegetative season: the start of the season (SOS), the flowering time at the middle of the season (MOS) and the end of the season (EOS).

The plant material harvested for both molecular and phytochemical analysis was immediately frozen in liquid nitrogen. Leaf samples were finely ground in mortar and pestle with liquid nitrogen. Root samples were finely ground using automatic electric grinder Analysette 3 Spartan and Pulverisette 0 Cryo-Box (Fritsch, GmbH, Idar-Oberstein, Germany) filled out with liquid nitrogen. All samples were stored at –80 °C until analyzed.

### Sample preparation for qualitative analysis

The solvents and other chemicals for extraction and chromatography were purchased from Merck (Darmstadt, Germany) and were of analytical or LC–MS grade. The samples for qualitative profiling were prepared as previously published^28^.

### Liquid Chromatography–Mass Spectrometry analysis

Ultra-high performance liquid chromatography quadrupole time of flight mass spectrometry (UHPLC-QTOF-MS) was carried out using Dionex Ultimate 3000 RS (Thermo Fisher Scientific,Waltham, MA, USA) chromatographic system coupled to a Bruker Compact (Bruker, Billerica, MA, USA) quadrupole time of flight (QTOF) mass spectrometer.

The detailed system settings and chromatography-mass spectrometry analysis conditions have been described previously^28^. Quantitative analysis using DAD-HPLC was performed using cryptotanshinone (CAS 35825–57-1, purity > 99% by HPLC, isolated in-house from the roots of *S. yangii*, and identified using NMR spectroscopy and compared to the authentic standard (PhytoLab, Vestenbergsgreuth, Germany), Supplementary Fig. S1–S4 and Table S10).

Calibration curve was constructed from 7-point linear concentration range from 700 to 5 µg/mL (r2 ≥ 0.996) of cryptotanshinone. Each of the 7 calibration levels was analyzed 3 times. Quantification was conducted based on peak areas acquired at λ = 254 nm. The concentration of compounds was expressed in milligram per gram dry weight (mg/g).

### RNA extraction and synthesis of cDNA libraries

Total RNA from leaves and roots of *S. abrotanoides* and *S. yangii* was isolated with Plant/Fungi Total RNA Purification Kit (Norgen Biotek Corp., Thorold, Canada).

#### Construction of cDNA libraries for RNA sequencing

Total RNA from leaves and roots of *S. abrotanoides* and *S. yangii* harvested in the middle of season (MOS) was used for the construction of cDNA libraries. The concentration, quality and integrity of total RNA were assessed by the 2100 Bioanalyzer system (Agilent Technologies, Santa Clara, CA, USA). Eight cDNA libraries were synthetized with NEBNext Ultra II Directional RNA Library Prep Kit for Illumina and indexed with NEBNext Multiplex Oligos for Illumina (New England Biolabs). The quality and concentration of cDNA libraries were assessed by the 2100 Bioanalyzer system with using the High Sensitivity DNA Assay (Agilent Technologies, Santa Clara, CA, USA). The established concentration of eight cDNA libraries were as follows: 3.86 ng/µl (*S. abrotanoides* leaves rep1), 4.47 ng/µl (*S. abrotanoides* leaves rep2), 18.42 ng/µl (*S. abrotanoides* roots rep1), 14.95 ng/µl (*S. abrotanoides* roots rep2), 9.05 ng/µl (*S. yangii* leaves rep1), 8.29 ng/µl (*S. yangii* leaves rep2), 15.36 ng/µl (*S. yangii* roots rep1), and 14.08 ng/µl (*S. yangii* roots rep2). All cDNA libraries had a volume of 19 µl.

#### cDNA synthesis for qualitative real-time RT-PCR analysis

Total RNA from leaves and roots of *S. abrotanoides* and *S. yangii* harvested in the start of season (SOS), the middle of season (MOS) and at the end of season (EOS) was used for cDNA synthesis. Single-strand cDNA was synthesized from Turbo DNA-*free*™ (Thermo Fisher Scientific,Waltham, MA, USA) treated RNA using SuperScript® IV (Thermo Fisher Scientific, Waltham, MA, USA) reverse transcriptase with oligo(dT)18 primer (Meridian Bioscience, Cincinnati, OH, USA). The assessment of cDNA quantity and quality was performed spectrophotometrically by the NanoDrop ™ 2000c (Thermo Fisher Scientific, Waltham, MA, USA), by the 2100 Bioanalyzer system and in real-time PCR.

### RNA sequencing and de novo assembly of transcriptomes

RNA sequencing was done by Macrogen Inc., (Korea) on Illumina HiSeq 2500; paired-end read: 2 × 100 bp. The raw paired-end reads were checked by FastQC software (www.bioinformatics.babraham.ac.uk), and the Trimmomatic tool^84^ was used to cut adaptors from reads, exclude short artifact sequences (< 50 bp) and remove low-quality sequences (with Phred score < 20). Next, trimmed reads from both species were transferred separately to de novo assembler to create *S. abrotanoides* and *S. yangii* transcriptomes. Assembly process was made by Trinity^85^ software ver. 2.1.1; with a k-mer size equal to 25 using the server (23 cores/120 GB RAM) of the Regional IT Centre (Olsztyn, Poland). To create a ‘reference *Perovskia* transcriptome’, the obtained transcripts were assembled (using Inchworm, Trinity module), and next divided into clusters, within which the de Bruijn graph was constructed (Chrysalis, Trinity module) and paralogical sequences were assigned (Butterfly, Trinity module). The reference transcriptome had gene-transcript structure with unigene as basic unit and transcript sequences as subunit. The transcriptomes were evaluated by the Benchmarking Universal Single-Copy Orthologs (BUSCO v.5.4.3) and TrinityStats.pl software, and additional sequences of both datasets were compared. The probe of 1000 transcripts from both datasets were blasted to calculate the pairwise identity for homology transcripts. The similarity of both transcriptomes allowed to select the transcriptome of *S. abrotanoides* as a reference transcriptome for differentially analyses. The *S. abrotanoides* transcriptome was annotated using Trinotate software and UniProt, SwissProt, Pfam and Rfam databases.

### Differential expression (DE) of transcripts and GO-enrichment analysis

To perform expression profiling of RNA extracted from roots and leaves from both plant species, the raw paired-end reads of each sample were realigned to the *S. abrotanoides* transcriptome using Bowtie tool^86^. Read counts for unigenes were calculated by RSEM software^87^ and normalized expression values were reported as fragments per kilobase of transcript per million mapped reads (FPKM).

The following four differential expression analyses were performed: (1) *S. abrotanoides* roots vs. *S. yangii* roots, (2) *S. abrotanoides* leaves vs. *S. yangii* leaves, (3) *S. abrotanoides* leaves vs. roots, (4) *S. yangii* leaves vs. roots. For each comparison, the following cut-offs were used: p-adjusted < 0.05 and absolute value of logarithmic fold change > 1. Differentially expressed genes (DEGs) were functionally annotated with *Arabidopsis thaliana* as a reference using g.profiler (http://biit.cs.ut.ee/gprofiler/) software. All plots were created by R Bioconductor packages: pheatmap, ggplot2 and GOplot.

Gene Ontology (GO) (http://www.geneontology.org/) was further used to category the function of the transcripts, and the transcripts were assigned to biological functions in three main GO categories: biological process (BP), cellular component (CC) and molecular function (MF)^88^. Additionally, pathway analysis was performed to elucidate the significant pathways and processes of DEGs using Kyoto Encyclopedia of Gene and Genomes (KEGG) (http://www.genome.jp/kegg).

To identify candidate genes for enzymes in the diterpenoid pathway, the *S. abrotanoides* transcript sequences were used as a query to search against nucleotide sequences from various *Salvia* species at NCBI database (http://www.ncbi.nlm.nih.gov) using the BLASTx algorithm. An e-value cut-off of 10^–5^ was applied to the homologue recognition.

Cytochrome P450 sequences were analysed, annotated and systematically named by prof. David Nelson^59^.

### Amplification of partial coding sequence (CDS) of *CYP76AH89* and *CYP76AH80* in *S. yangii* and *S. abrotanoides*, Sanger sequencing and sequence analysis

Primers for amplification of *CYP76AH89* and *CYP76AH80* gene fragments were designed based on the sequences generated by RNA-sequencing (Supplementary Table S8). PCR reactions were performed as described earlier and PCR products were visualized by gel electrophoresis^25^. Amplified DNA products were purified with the Syngen Gel/PCR Mini Kit (Syngen, Wroclaw, Poland).

Sanger sequencing was carried out with the BrilliantDye™ Terminator v3.1 Kit (Nimagen B.V., Nijmegen, The Netherlands). Reactions were set with a fourfold dilution of the reaction premix and the addition of BrilliantDye® Terminator 5X Sequencing Buffer (Nimagen B.V., Nijmegen, The Netherlands) according to the producer’s instructions. Sequencing products were precipitated with ethanol, dissolved in TSR (Hi-Di Formamide) (Thermo Fisher Scientific, Waltham, MA, USA) and then separated by capillary electrophoresis on the Applied Biosystems™ 310 Genetic Analyzer (Thermo Fisher Scientific, Waltham, MA, USA). Capillary electrophoresis was performed in a 50 cm long capillary filled with POP-7 Polymer (Thermo Fisher Scientific, Waltham, MA, USA). The separation time was 1 h 40 min, and the run voltage was 6 kV with 60 sec and 2 kV sample injection. Two reads were collected for each sample. Two partial CDSs were generated for *S. abrotanoides* (ON365538, ON365540) and two for *S. yangii* (ON365539, ON365541).

The sequence quality was checked with the Sanger Quality Check App (Thermo Fisher Scientific,Waltham, MA, USA). Forward and reverse sequencing reads for each CDS were assembled into a contig using the BioEdit Sequence Alignment Editor^89^. The identity of all obtained sequences was confirmed through similarity to published sequences using the BLAST algorithm.

### Expression analysis of abietane diterpenoid pathway-related genes

The gene-specific primers listed in Supplementary Table S8 were used for the analysis of gene expression based on reverse-transcribed cDNA products as templates. The real-time PCR primers for genes encoding the downstream enzymes from the abietane diterpenoids biosynthesis pathway in *S. abrotanoides* and *S. yangii* were designed with Primer3Plus (http://www.bioinformatics.nl/primer3plus, accessed on 13 August 2021, 29 September 2021 and 11 April 2023) using the sequences generated by RNA-seq (BioProject accession number PRJNA773198)^90^. Each real-time PCR reaction was performed in triplicates. The instrumental settings and quantitative real-time PCR conditions were used as previously published^91^. The relative quantification using *actin* (*ACT*) genes (accession numbers MW240684 and MW240685) as a reference was used for the determination of the transcript levels. The stability of the reference gene was established with the RefFinder (https://blooge.cn/RefFinder/)^92^. Reaction efficiency for each pair of primers was estimated experimentally (by calibration curve) and used for calculations using the Pfaffl mathematical model^93^. All steps starting from experiment design through RNA isolation to data analysis were conducted according to the MIQE guidelines provided for quantitative real-time PCR analysis^94^. Normalized expression (NE) data are presented in Supplementary Table S7.

### Statistical data evaluation

A one-way ANOVA with post hoc Tukey’s multiple comparison tests (GraphPad Prism, San Diego, CA, USA) was used to determine the statistical significance of differences between phytochemical profiles and, in parallel, between means of expression data sets. Differences were regarded as significant at p < 0.05 (Supplementary Table S9).

### Supplementary Information

Supplementary Information 1.

Supplementary Table 2.

Supplementary Table 3.

Supplementary Table 4.

Supplementary Table 5.

Supplementary Table 6.

Supplementary Table 7.

Supplementary Table 8.

Supplementary Table 9.

## Data Availability

Generated RNA-seq data for *S. abrotanoides* and *S. yangii* has been deposited in the NCBI database (SRA; https://www.ncbi.nlm.nih.gov/sra) under BioProject accession number PRJNA773198. DNA sequence data generated in this study has been deposited in GeneBank at accession numbers: ON365538, ON365539, ON365540, ON365541.

## References

[1] Khaliq S, Volk FJ, Frahm AW (2007). Phytochemical investigation of *Perovskia abrotanoides*. Planta Med..

[2] Flora of Pakistan Available online: http://www.efloras.org/flora_page.aspx?flora_id=5, accessed 17 January 2020.

[3] Perveen S, Malik A, Tareen RB (2009). Structural determination of atricins A and B, new triterpenes from *Perovskia atriplicifolia*, by 1D and 2D NMR spectroscopy. Magn. Reson. Chem..

[4] Flora of China Available online: http://www.efloras.org/flora_page.aspx?flora_id=2, accessed 17 January 2020).

[5] Mohammadhosseini M, Venditti A, Akbarzadeh A (2019). The genus Perovskia Kar.: Ethnobotany, chemotaxonomy and phytochemistry: A review. Toxin Rev..

[6] Tareen RB, Bibi T, Khan MA, Ahmad M, Zafar M (2010). Indigenous knowledge of folk medicine by the women of Kalat and Khuzdar regions of Balochistan. Pakistan. Pakistan J. Bot..

[7] Phani Kumar P, Gupta S, Murugan M, Bala-Singh S (2009). Ethnobotanical studies of Nubra valley-A cold arid zone of Himalaya. Ethnobot Leafl..

[8] Moallem SA, Niapour M (2008). Study of embryotoxicity of *Perovskia abrotanoides*, an adulterant in folk-medicine, during organogenesis in mice. J. Ethnopharmacol..

[9] Ballabh B, Chaurasia OP, Ahmed Z, Singh SB (2008). Traditional medicinal plants of cold desert Ladakh-Used against kidney and urinary disorders. J. Ethnopharmacol..

[10] Hosseinzadeh, H.; Amel, S. Antinociceptive Effect of the Aerial Parts of *Perovskia brotanoides* Extracts in Mice. *Iran. Red Crescent Med. J.* **2009**, *6*.

[11] Sairafianpour M, Christensen J, Steerk D, Budnik BA, Kharazmi A, Bagherzadeh K, Jaroszewski JW (2001). Leishmanicidal, antiplasmodial, and cytotoxic activity of novel diterpenoid 1,2-quinones from *Perovskia abrotanoides*: New source of tanshinones. J. Nat. Prod..

[12] Jaafari MR, Hooshmand S, Samiei A, Hossainzadeh H (2007). Evaluation of leishmanicidal effect of *Perovskia abrotanoides* Karel. Root extract by in vitro leishmanicidal assay using promastigotes of Leishmania major. Pharmacologyonline.

[13] Perveen S, Malik A, Noor AT, Tareen RB (2008). Pervosides A and B, new isoferulyl glucosides from *Perovskia atriplicifolia*. J. Asian Nat. Prod. Res..

[14] Baquar SR (1989). Medicinal and poisonous plants of Pakistan.

[15] Eisenman, S.W.; Zaurov, D.E.; Struwe, L. *Medicinal plants of Central Asia: Uzbekistan and Kyrgyzstan*; 2013

[16] Gao L, Zhou J, Zhu LY, Zhang JR, Jing YX, Zhao JW, Huang XZ, Li GP, Jiang ZY, Xue DY (2017). Four new diterpene glucosides from *Perovskia atriplicifolia*. Chem. Biodivers..

[17] Jiang ZY, Yu YJ, Huang CG, Huang XZ, Hu QF, Yang GY, Wang HB, Zhang XY, Li GP (2015). Icetexane diterpenoids from *Perovskia atriplicifolia*. Planta Med..

[18] Sajjadi SE, Mehregan I, Khatamsaz M, Asgari G (2005). Chemical composition of the essential oil of *Perovskia abrotanoides* Karel. growing wild in Iran. Flavour Fragr. J..

[19] Jassbi AR, Ahmad VU, Tareen RB (1999). Constituents of the essential oil of *Perovskia atriplicifolia*. Flavour Fragr. J..

[20] Erdemgil FZ, Ilhan S, Korkmaz F, Kaplan C, Mercangöz A, Arfan M, Ahmad S (2007). Chemical composition and biological activity of the essential oil of *Perovskia atriplicifolia* from Pakistan. Pharm. Biol..

[21] Dabiri M, Sefidkon F (2001). Analysis of the essential oil from aerial parts of *Perovskia atriplicifolia* Benth. at different stages of plant growth. Flavour Fragr. J..

[22] Perveen S, Khan SB, Malik A, Tareen RB, Nawaz SA, Choudhary MI (2006). Phenolic constituents from *Perovskia atriplicifolia*. Nat. Prod. Res..

[23] Tarawneh A, León F, Pettaway S, Elokely KM, Klein ML, Lambert J, Mansoor A, Cutler SJ (2015). Flavonoids from *Perovskia atriplicifolia* and their in vitro displacement of the respective radioligands for human opioid and cannabinoid receptors. J. Nat. Prod..

[24] Ghaderi S, Nejad Ebrahimi S, Ahadi H, Eslambolchi Moghadam S, Mirjalili MH (2019). In vitro propagation and phytochemical assessment of *Perovskia abrotanoides* Karel. (Lamiaceae)—A medicinally important source of phenolic compounds. Biocatal. Agric. Biotechnol..

[25] Stafiniak M, Ślusarczyk S, Pencakowski B, Matkowski A, Rahimmalek M, Bielecka M (2021). Seasonal variations of rosmarinic acid and its glucoside and expression of genes related to their biosynthesis in two medicinal and aromatic species of *Salvia* subg. *Perovskia*. Biology (Basel)..

[26] Božić D, Papaefthimiou D, Brückner K, De Vos RCH, Tsoleridis CA, Katsarou D, Papanikolaou A, Pateraki I, Chatzopoulou FM, Dimitriadou E (2015). Towards elucidating carnosic acid biosynthesis in Lamiaceae: Functional characterization of the three first steps of the pathway in *Salvia fruticosa* and *Rosmarinus officinalis*. PLoS One.

[27] Jassbi AR, Zare S, Firuzi O, Xiao J (2016). Bioactive phytochemicals from shoots and roots of Salvia species. Phytochem. Rev..

[28] Bielecka M, Pencakowski B, Stafiniak M, Jakubowski K, Rahimmalek M, Gharibi S, Matkowski A, Ślusarczyk S (2021). Metabolomics and DNA-based authentication of two traditional Asian medicinal and aromatic species of *Salvia* subg. *Perovskia*. Cells.

[29] Ge X, Wu J (2005). Tanshinone production and isoprenoid pathways in *Salvia miltiorrhiza* hairy roots induced by Ag + and yeast elicitor. Plant Sci..

[30] Lu, S. *Compendium of Plant Genomes*; Springer Nature Switzerland AG, 2019; ISBN 978-3-030-24715-7.

[31] Wang JW, Wu JY (2010). Tanshinone biosynthesis in *Salvia miltiorrhiza* and production in plant tissue cultures. Appl. Microbiol. Biotechnol..

[32] Su CY, Ming QL, Rahman K, Han T, Qin LP (2015). *Salvia miltiorrhiza*: Traditional medicinal uses, chemistry, and pharmacology. Chin. J. Nat. Med..

[33] Song JJ, Fang X, Li CY, Jiang Y, Li JX, Wu S, Guo J, Liu Y, Fan H, Huang YB (2022). A 2-oxoglutarate-dependent dioxygenase converts dihydrofuran to furan in Salvia diterpenoids. Plant Physiol..

[34] Wang X, Morris-Natschke SL, Lee KH (2007). New developments in the chemistry and biology of the bioactive constituents of Tanshen. Med. Res. Rev..

[35] Mei XD, Cao YF, Che YY, Li J, Shang ZP, Zhao WJ, Qiao YJ, Zhang JY (2019). Danshen: a phytochemical and pharmacological overview. Chin. J. Nat. Med..

[36] González MA (2015). Aromatic abietane diterpenoids: their biological activity and synthesis. Nat. Prod. Rep..

[37] Ma Y, Yuan L, Wu B, Li X, Chen S, Lu S (2012). Genome-wide identification and characterization of novel genes involved in terpenoid biosynthesis in *Salvia miltiorrhiza*. J. Exp. Bot..

[38] Cui G, Huang L, Tang X, Zhao J (2011). Candidate genes involved in tanshinone biosynthesis in hairy roots of *Salvia miltiorrhiza* revealed by cDNA microarray. Mol. Biol. Rep..

[39] Yang L, Ding G, Lin H, Cheng H, Kong Y, Wei Y, Fang X, Liu R, Wang L, Chen X (2013). Transcriptome analysis of medicinal plant *Salvia miltiorrhiza* and identification of genes related to tanshinone biosynthesis. PLoS One.

[40] Gao W, Sun H-X, Xiao H, Cui G, Hillwig ML, Jackson A, Wang X, Shen Y, Zhao N, Zhang L (2014). Combining metabolomics and transcriptomics to characterize tanshinone biosynthesis in *Salvia miltiorrhiza*. BMC Genomics.

[41] Cui G, Duan L, Jin B, Qian J, Xue Z, Shen G, Snyder JH, Song J, Chen S, Huang L (2015). Functional divergence of diterpene syntheses in the medicinal plant *Salvia miltiorrhiza*. Plant Physiol..

[42] Brückner K, Božić D, Manzano D, Papaefthimiou D, Pateraki I, Scheler U, Ferrer A, De Vos RCH, Kanellis AK, Tissier A (2014). Characterization of two genes for the biosynthesis of abietane-type diterpenes in rosemary (*Rosmarinus officinalis*) glandular trichomes. Phytochemistry.

[43] Trikka FA, Nikolaidis A, Ignea C, Tsaballa A, Tziveleka L-A, Ioannou E, Roussis V, Stea EA, Božić D, Argiriou A (2015). Combined metabolome and transcriptome profiling provides new insights into diterpene biosynthesis in *S. pomifera* glandular trichomes. BMC Genomics.

[44] Hu J, Wang F, Liang F, Wu Z, Jiang R, Li J, Chen J, Qiu S, Wang J, Zhang Y (2022). Identification of abietane-type diterpenoids and phenolic acids biosynthesis genes in *Salvia apiana* Jepson through full-length transcriptomic and metabolomic profiling. Front. Plant Sci..

[45] Wang Z, Peters RJ (2022). Tanshinones: Leading the way into Lamiaceae labdane-related diterpenoid biosynthesis. Curr. Opin. Plant Biol..

[46] Vranová E, Coman D, Gruissem W (2013). Network analysis of the MVA and MEP pathways for isoprenoid synthesis. Ann. Rev. Plant. Biol..

[47] Peters RJ (2010). Two rings in them all: The labdane-related diterpenoids. Nat. Prod. Rep..

[48] Gao W, Hillwig ML, Huango L, Cui G, Wang X, Kong J, Yang B, Peters RJ (2009). A functional genomics approach to tanshinone biosynthesis provides stereochemical insights. Org. Lett..

[49] Guo J, Zhou YJ, Hillwig ML, Shen Y, Yang L, Wang Y, Zhang X, Liu W, Peters RJ, Chen X (2013). CYP76AH1 catalyzes turnover of miltiradiene in tanshinones biosynthesis and enables heterologous production of ferruginol in yeasts. Proc. Natl. Acad. Sci. USA.

[50] Zi J, Peters RJ (2013). Characterization of CYP76AH4 clarifies phenolic diterpenoid biosynthesis in the Lamiaceae. Org. Biomol. Chem..

[51] Ma Y, Ma X-H, Meng F-Y, Zhan Z-L, Guo J, Huang L-Q (2016). RNA interference targeting CYP76AH1 in hairy roots of *Salvia miltiorrhiza* reveals its key role in the biosynthetic pathway of tanshinones. Biochem. Biophys. Res. Commun..

[52] Guo J, Ma X, Cai Y, Ma Y, Zhan Z, Zhou YJ, Liu W, Guan M, Yang J, Cui G (2016). Cytochrome P450 promiscuity leads to a bifurcating biosynthetic pathway for tanshinones. New Phytol..

[53] Ma Y, Cui G, Chen T, Ma X, Wang R, Jin B, Yang J, Kang L, Tang J, Lai C (2021). Expansion within the CYP71D subfamily drives the heterocyclization of tanshinones synthesis in *Salvia miltiorrhiza*. Nat. Commun..

[54] Xu Z, Peters RJ, Weirather J, Luo H, Liao B, Zhang X, Zhu Y, Ji A, Zhang B, Hu S (2015). Full-length transcriptome sequences and splice variants obtained by a combination of sequencing platforms applied to different root tissues of *Salvia miltiorrhiza* and tanshinone biosynthesis. Plant J..

[55] Birtić S, Dussort P, Pierre FX, Bily AC, Roller M (2015). Carnosic acid. Phytochemistry.

[56] Loussouarn M, Krieger-Liszkay A, Svilar L, Bily A, Birtić S, Havaux M (2017). Carnosic acid and carnosol, two major antioxidants of rosemary, act through different mechanisms. Plant Physiol..

[57] Ignea C, Athanasakoglou A, Ioannou E, Georgantea P, Trikka FA, Loupassaki S, Roussis V, Makris AM, Kampranis SC (2016). Carnosic acid biosynthesis elucidated by a synthetic biology platform. Proc. Natl. Acad. Sci. U. S. A..

[58] Scheler U, Brandt W, Porzel A, Rothe K, Manzano D, Bozic D, Papaefthimiou D, Balcke GU, Henning A, Lohse S (2016). Elucidation of the biosynthesis of carnosic acid and its reconstitution in yeast. Nat. Commun..

[59] Nelson DR (2009). The cytochrome P450 homepage. Hum. Genomics.

[60] Mao Y, Ma Y, Chen T, Ma X, Xu Y, Bu J, Li Q, Jin B, Wang Y, Li Y (2020). Functional integration of two CYP450 genes involved in biosynthesis of tanshinones for improved diterpenoid production by synthetic biology. ACS Synth. Biol..

[61] Hu Z, Liu X, Tian M, Ma Y, Jin B, Gao W, Cui G, Guo J, Huang L (2021). Recent progress and new perspectives for diterpenoid biosynthesis in medicinal plants. Med. Res. Rev..

[62] Lanier ER, Andersen TB, Hamberger B (2023). Plant terpene specialized metabolism: Complex networks or simple linear pathways?. Plant J..

[63] Werck-Reichhart D (2023). Promiscuity, a driver of plant cytochrome P450 evolution?. Biomolecules.

[64] Bryson AE, Lanier ER, Lau KH, Hamilton JP, Vaillancourt B, Mathieu D, Yocca AE, Miller GP, Edger PP, Buell CR (2023). Uncovering a miltiradiene biosynthetic gene cluster in the Lamiaceae reveals a dynamic evolutionary trajectory. Nat. Commun..

[65] Su Y, Zhang J, Xu Z, Li J, Wang P, Song Z, Tian G, Li L, Song J, Wang J (2021). Integrative analysis of metabolome and transcriptome reveals the mechanism of color formation in white root (*Salvia miltiorrhiza*). Ind. Crops Prod..

[66] Li B, Cui G, Shen G, Zhan Z, Huang L, Chen J, Qi X (2017). Targeted mutagenesis in the medicinal plant *Salvia miltiorrhiza*. Sci. Rep..

[67] Li B, Li J, Chai Y, Huang Y, Li L, Wang D, Wang Z (2021). Targeted mutagenesis of CYP76AK2 and CYP76AK3 in *Salvia miltiorrhiza* reveals their roles in tanshinones biosynthetic pathway. Int. J. Biol. Macromol..

[68] Zhao JL, Zhou LG, Wu JY (2010). Effects of biotic and abiotic elicitors on cell growth and tanshinone accumulation in *Salvia miltiorrhiza* cell cultures. Appl. Microbiol. Biotechnol..

[69] Xing B, Yang D, Guo W, Liang Z, Yan X, Zhu Y, Liu Y (2015). Ag+ as a more effective elicitor for production of tanshinones than phenolic acids in *Salvia miltiorrhiza* hairy roots. Molecules.

[70] Wang CH, Zheng LP, Tian H, Wang JW (2016). Synergistic effects of ultraviolet-B and methyl jasmonate on tanshinone biosynthesis in *Salvia miltiorrhiza* hairy roots. J. Photochem. Photobiol. B Biol..

[71] Shi M, Kwok KW, Wu JY (2007). Enhancement of tanshinone production in *Salvia miltiorrhiza* Bunge (red or Chinese sage) hairy-root culture by hyperosmotic stress and yeast elicitor. Biotechnol. Appl. Biochem..

[72] Shi M, Hua Q, Kai G (2021). Comprehensive transcriptomic analysis in response to abscisic acid in *Salvia miltiorrhiza*. Plant Cell. Tissue Organ Cult..

[73] Contreras A, Leroy B, Mariage PA, Wattiez R (2019). Proteomic analysis reveals novel insights into tanshinones biosynthesis in *Salvia miltiorrhiza* hairy roots. Sci. Rep..

[74] Hou Z, Li Y, Su F, Wang Y, Zhang X, Xu L, Yang D, Liang Z (2021). The exploration of methyl jasmonate on the tanshinones biosynthesis in hair roots of *Salvia miltiorrhiza* Bunge and *Salvia castanea* f. *tomentosa* Stib. Ind. Crops Prod..

[75] Jiang Y, Wang L, Lu S, Xue Y, Wei X, Lu J, Zhang Y (2019). Transcriptome sequencing of *Salvia miltiorrhiza* after infection by its endophytic fungi and identification of genes related to tanshinone biosynthesis. Pharm. Biol..

[76] Jiang HL, Wang XZ, Xiao J, Luo XH, Yao XJ, Zhao YY, Chen YJ, Crews P, Wu QX (2013). New abietane diterpenoids from the roots of *Salvia przewalskii*. Tetrahedron.

[77] Ulubelen A (1990). New diterpenoids from the roots of *Salvia triloba*. Planta Med..

[78] Slusarczyk S, Topolski J, Domaradzki K, Adams M, Hamburger M, Matkowski A (2015). Isolation and fast selective determination of nor-abietanoid diterpenoids from *Perovskia atriplicifolia* roots using LC-ESI-MS/MS with multiple reaction monitoring. Nat. Prod. Commun..

[79] Wang Q, Hillwig ML, Okada K, Yamazaki K, Wu Y, Swaminathan S, Yamane H, Peters RJ (2012). Characterization of CYP76M5-8 indicates metabolic plasticity within a plant biosynthetic gene cluster. J. Biol. Chem..

[80] Teimoori-Boghsani Y, Ganjeali A, Cernava T, Müller H, Asili J, Berg G (2020). Endophytic fungi of native *Salvia abrotanoides* plants reveal high taxonomic diversity and unique profiles of secondary metabolites. Front. Microbiol..

[81] Ma C, Jiang D, Wei X (2011). Mutation breeding of *Emericella foeniculicola* TR21 for improved production of tanshinone IIA. Process Biochem..

[82] Ming Q, Han T, Li W, Zhang Q, Zhang H, Zheng C, Huang F, Rahman K, Qin L (2012). Tanshinone IIA and tanshinone i production by *Trichoderma atroviride* D16, an endophytic fungus in *Salvia miltiorrhiza*. Phytomedicine.

[83] Sagita R, Quax WJ, Haslinger K (2021). Current state and future directions of genetics and genomics of endophytic fungi for bioprospecting efforts. Front. Bioeng. Biotechnol..

[84] Bolger AM, Lohse M, Usadel B (2014). Trimmomatic: A flexible trimmer for Illumina sequence data. Bioinformatics.

[85] Grabherr MG, Haas BJ, Yassour M, Levin JZ, Thompson DA, Amit I, Adiconis X, Fan L, Raychowdhury R, Zeng Q (2011). Full-length transcriptome assembly from RNA-Seq data without a reference genome. Nat. Biotechnol..

[86] Langmead B, Trapnell C, Pop M, Salzberg SL (2009). Ultrafast and memory-efficient alignment of short DNA sequences to the human genome. Genome Biol..

[87] Li B, Dewey CN (2011). RSEM: Accurate transcript quantification from RNA-Seq data with or without a reference genome. BMC Bioinform.

[88] Reimand J, Kull M, Peterson H, Hansen J, Vilo J (2007). g:Profiler—a web-based toolset for functional profiling of gene lists from large-scale experiments. Nucleic Acids Res..

[89] Hall TA (1999). BioEdit: A user-friendly biological sequence alignment editor and analysis program for Windows 95/98/NT. Nucleic Acids Symp. Ser..

[90] Untergasser A, Nijveen H, Rao X, Bisseling T, Geurts R, Leunissen JAM (2007). Primer3Plus, an enhanced web interface to Primer3. Nucleic Acids Res..

[91] Bielecka M, Zielińska S, Pencakowski B, Stafiniak M, Ślusarczyk S, Prescha A, Matkowski A (2019). Age-related variation of polyphenol content and expression of phenylpropanoid biosynthetic genes in *Agastache rugosa*. Ind. Crops Prod..

[92] Xie F, Wang J, Zhang B (2023). RefFinder: A web-based tool for comprehensively analyzing and identifying reference genes. Funct. Integr. Genomics.

[93] Pfaffl MW (2001). A new mathematical model for relative quantification in real-time RT-PCR. Nucleic Acids Res..

[94] Bustin SA, Benes V, Garson JA, Hellemans J, Huggett J, Kubista M, Mueller R, Nolan T, Pfaffl MW, Shipley GL (2009). The MIQE guidelines: Minimum information for publication of quantitative real-time PCR experiments. Clin. Chem..

