# Supplementary material for: Comparative transcriptomics of two Salvia subg. Perovskia species contribute towards molecular background of abietane-type diterpenoid biosynthesis
Source: Sci Rep. 2024 Feb 6;14:3046. doi: 10.1038/s41598-024-53510-5 (PMC10847172; doi:10.1038/s41598-024-53510-5)
Supplement: Supplementary file 1 — Supplementary Information 1. [file 41598_2024_53510_MOESM1_ESM.docx]

Fig S1. Calibration curve for cryptotanshinone

**Table S10.** ^1^H-NMR (500 MHz) and ^13^C-NMR of cryptotanshinone in CDCl_3_

| **Nb** | δ _H_ (*J* w Hz) | δ _C_*^a^* |  |
| --- | --- | --- | --- |
| **1** | 3.19 *tr* (5.7) | 29.7 |  |
| **2** | 1.77 *m* | 19.1 |  |
| **3** | 1.63 *m* | 37.1 |  |
| **4** |  | 34.9 |  |
| **5** |  | 152.2 |  |
| **6** | 7.59 *d* (9.6) | 132.4 |  |
| **7** | 7.46 *d* (9.6) | 122.5 |  |
| **8** |  | 126.3 |  |
| **9** |  | 128.2 |  |
| **10** |  | 143.7 |  |
| **11** |  | 184.2 |  |
| **12** |  | 175.7 |  |
| **13** |  | 118.3 |  |
| **14** |  | 170.7 |  |
| **15** | 4.85 *dd* (9.8, 6.6), 4.32 *dd* (9.8, 6.6) | 81.4 |  |
| **16** | 3.56 *m* (6.9) | 34.6 |  |
| **17** | 1.1 *d* (6.7) | 18.7 |  |
| **18** | 1.28 *s* | 31.9 |  |
| **19** | 1.28 *s* | 31.9 |  |

*^a^*^13^C (based on 2D HSQC and HMBC-NMR)

^b^ δ_H,_ δ_C -_ Shaik, F. H., *Beilstein journal of organic chemistry*, 2009, 47,
Lee, S.-Y. *Archives of pharmacal research*, 2005, 28(8), 909–13,
Sairafianpour, M. *Journal of natural products*, 2001, 64(11), 1398–1403

**4,4,16-trimethyl-1,2,3,4,15,16-heksahydrophenatro[13,14-*b*]furan-11,12-dione (16R)**

CAS Registry Number: 35825-57-1

Synonyms: Cryptotanshinone, Cryptotanshinon, Tanshinone c

C_19_H_20_O_3 ­_M-296.36

*m/z* (ESI-TOF-MS) 319.1367 [M+Na]^+^ calculated 319.1310

Fig S2. ^1^H-NMR

**Fig. S3.** 2D-COS-NMR

**Fig S4.** 2D-HSQC-NMR
